# *N*-Hydroxypiridinedione: A Privileged Heterocycle for Targeting the HBV RNase H

**DOI:** 10.3390/molecules29122942

**Published:** 2024-06-20

**Authors:** Dimitrios Moianos, Maria Makri, Georgia-Myrto Prifti, Aristeidis Chiotellis, Alexandros Pappas, Molly E. Woodson, Razia Tajwar, John E. Tavis, Grigoris Zoidis

**Affiliations:** 1 Division of Pharmaceutical Chemistry, Department of Pharmacy, School of Health Sciences, National and Kapodistrian University of Athens, Panepistimiopolis Zografou, 15771 Athens, Greece; moianosjim@pharm.uoa.gr (D.M.); mmakri@pharm.uoa.gr (M.M.); geprifti@pharm.uoa.gr (G.-M.P.); 2 Institute of Nuclear & Radiological Sciences & Technology, Energy & Safety, National Center for Scientific Research “Demokritos”, 15310 Athens, Greece; achiotel@rrp.demokritos.gr (A.C.); alpappas@pharm.uoa.gr (A.P.); 3 Molecular Microbiology and Immunology, Saint Louis University School of Medicine, Saint Louis, MO 63104, USA; molly.woodson@slu.edu (M.E.W.); razia.tajwar@health.slu.edu (R.T.); john.tavis@health.slu.edu (J.E.T.)

**Keywords:** hepatitis B virus, ribonuclease H, *N*-hydroxypyridinediones, oximes, structure–activity relationships, anti-HBV agents

## Abstract

Hepatitis B virus (HBV) remains a global health threat. Ribonuclease H (RNase H), part of the virus polymerase protein, cleaves the pgRNA template during viral genome replication. Inhibition of RNase H activity prevents (+) DNA strand synthesis and results in the accumulation of non-functional genomes, terminating the viral replication cycle. RNase H, though promising, remains an under-explored drug target against HBV. We previously reported the identification of a series of *N*-hydroxypyridinedione (HPD) imines that effectively inhibit the HBV RNase H. In our effort to further explore the HPD scaffold, we designed, synthesized, and evaluated 18 novel HPD oximes, as well as 4 structurally related minoxidil derivatives and 2 barbituric acid counterparts. The new analogs were docked on the RNase H active site and all proved able to coordinate the two Mg^2+^ ions in the catalytic site. All of the new HPDs effectively inhibited the viral replication in cell assays exhibiting EC_50_ values in the low μM range (1.1–7.7 μM) with low cytotoxicity, resulting in selectivity indexes (SI) of up to 92, one of the highest reported to date among HBV RNase H inhibitors. Our findings expand the structure–activity relationships on the HPD scaffold, facilitating the development of even more potent anti-HBV agents.

## 1. Introduction

Hepatitis B virus (HBV) poses a severe health burden worldwide. While a safe and effective HBV vaccine exists, current estimates reveal 1.5 million new infections per year around the globe and 296 million chronic HBV patients, resulting in 820,000 deaths annually [1,2].

Current treatments for HBV infection use two primary compound categories: nucleos(t)ide analogs (NAs) and pegylated interferon alpha [3,4]. Interferon alpha treatment use is limited due to its subcutaneous injection and serious side effects including flu-like symptoms, bone marrow suppression, fatigue, and depression, which also decrease the patient adherence to the treatment [5]. NAs, mainly represented by entecavir, tenofovir disoproxil fumarate, and tenofovir alafenamide, inhibit the reverse transcriptase (RT) enzymatic activity of the viral polymerase (P) protein. They are administered orally, have an excellent safety profile, and effectively suppress HBV replication, showing a high antiviral potency. Nevertheless, HBsAg clearance is achieved in only 3–5% of patients, after 10 years of NA treatment [6]. Since NAs induce profound but incomplete suppression of HBV DNA replication but have no direct effect on the viral cccDNA that templates all viral transcripts, drug administration is often life-long and viral replication usually resurges on treatment cessation [7,8]. Considering the limitations of existing HBV treatments, there is a compelling need to develop drugs that employ alternative stages in the virus’s replication cycle [9,10,11,12,13].

HBV replicates via reverse transcription. The viral polymerase (P) protein has two distinct enzymatic domains, the RT and ribonuclease H (RNase H). The RT catalyzes synthesis of the negative polarity DNA strand [(−) DNA] using the virus pregenomic RNA (pgRNA) as a template. Once the (−) DNA strand has been synthesized inside the viral capsid, the RNase H domain cleaves the pgRNA template strand to expose the newly synthesized (−) DNA strand so it can template synthesis of the (+) DNA strand [14,15]. RNase H is a metalloenzyme and acts through a metal-chelation hydrolysis mechanism which requires two Mg^2+^ ions coordinated by four carboxylic amino acid moieties (the “D-E-D-D” motif) in the enzyme‘s active site [16]. There is no crystal structure for HBV P or its RNase H domain, so our group generated and validated an HBV P folding model (including the RNase H catalytic domain) using AlphaFold [17]. AlphaFold is an AI system from Google DeepMind that can reliably predict the 3D structure of a protein from its amino acid sequence, often achieving an accuracy comparable to experimental methods [18]. This model accurately predicts the essential role the two Mg^2+^ ions play during the pgRNA hydrolysis mechanism, enabling the application of computational chemistry techniques, for the development of potential RNase H inhibitors [18]. Inhibition of the RNase H enzymatic activity prevents the synthesis of (+) DNA and therefore the synthesis of functional viral genomes. Non-functional RNA:DNA heteroduplexes accumulate in the newly synthesized nucleocapsids, terminating viral replication and cccDNA maintenance [19]. The HBV RNase H is not targeted by any of the current anti-HBV drugs.

**Figure 1:**
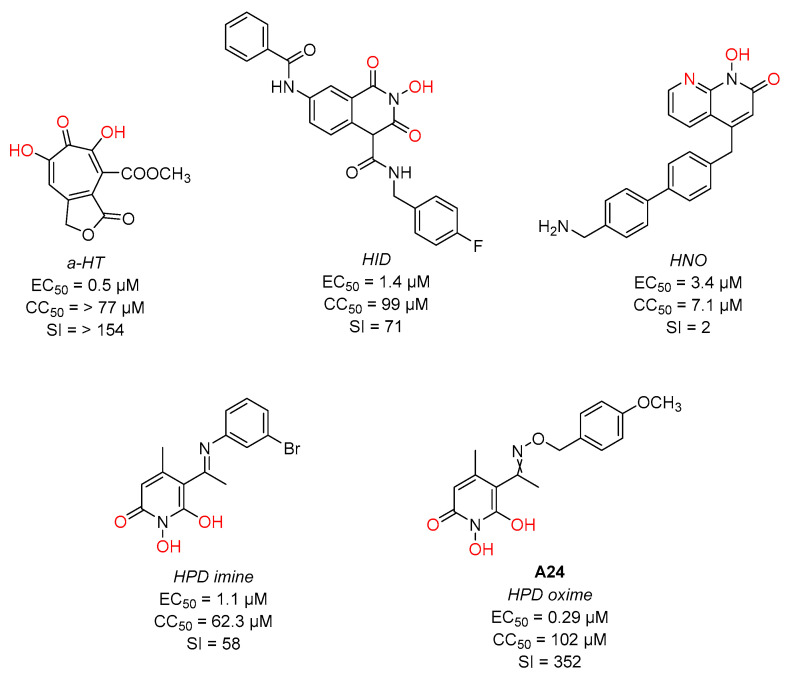
Chemical structures and biological activity of potent example HBV RNase H inhibitors from each chemotype. α-HT: α-Hydroxytropolone; HID: *N*-Hydroxyisoquinolinedione; HNO: *N*-Hydroxynapthyridinone; HPD: *N*-Hydroxypyridinedione [21,23,28,29]. **A24** is an approximately equimolar mixture of the E/Z isomers. EC_50_, effective concentration 50%; CC_50_, cytotoxic concentration 50%; SI, selectivity index (CC_50_/EC_50_).

Previously, our group reported several potent HBV RNase H inhibitors belonging to four chemical classes: α-hydroxytropolones (αHTs), *N*-hydroxyisoqinolinediones (HIDs), *N*-hydroxypyridinediones (HPDs), and *N*-hydroxynapthyridinones (HNOs) (Figure 1) [19,20,21,22,23,24,25,26,27]. All inhibitors of HBV RNase H possess three electron donors (either O or N atoms) in appropriate positions for chelating the two Mg^2+^ ions in the enzyme’s catalytic site [28]. Among the four inhibitor chemical categories, HPDs have demonstrated the most promising results in terms of inhibiting HBV replication, as well as having favorable druglike properties [21,29,30]. We have designed, synthesized, and tested HPDs belonging to two distinct chemical groups: imine HPDs and oxime HPDs. Recently, as part of our ongoing efforts to develop even more potent HBV RNase H inhibitors, we set out to further explore the HPD imine scaffold. We identified several novel potent HPD imines with EC_50_ values as low as 1.1 μΜ and selectivity indexes (SI) of up to 58. These findings represented a substantial improvement over the previously reported most potent HPD imine [30].

**Figure 2:**
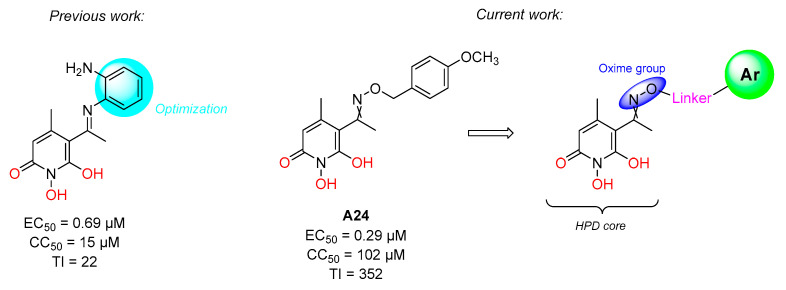
Design of *N*-hydroxypyridinediones (HPDs) oximes: optimization of the oxime side substitution.

Here, we implemented a medicinal chemistry approach aiming at refining the HPD oxime scaffold. Our goal was to deepen our understanding of the structure–activity relationships and to further improve the potency, toxicity, and druglike properties of this compound chemotype. We designed and synthesized 18 novel HPD oximes, leaving the main scaffold (which contains the O “trident” that chelates with the Mg^2+^ ions) intact and modifying the side chain. We used A24, the most promising HPD oxime (in terms of SI value, SI = 352 [29]) as our lead compound, and we developed HPDs mostly bearing an aromatic side substitution (Figure 2). We also synthesized HPD oxime analogs with two aromatic side rings as well as alkynes as a side substitution. Additionally, we altered the linker between the main HPD scaffold and the side group. Furthermore, to explore the role of the oxygen “trident” in activity, we synthesized two structurally related barbituric acid analogs and four analogs bearing the main scaffold of the known drug minoxidil. The minoxidil main pyrimidine ring contains three atoms (two N and one O atom) in suitable positions to chelate the Mg^2+^ ions of the enzyme active site and has already been proved able to chelate divalent cations [31,32]. All new compounds were computationally docked in the RNase H active site of the P structural model and evaluated in cell assays for their ability to inhibit HBV replication.

## 2. Results and Discussion

### 2.1. Chemistry

**Scheme 1:**
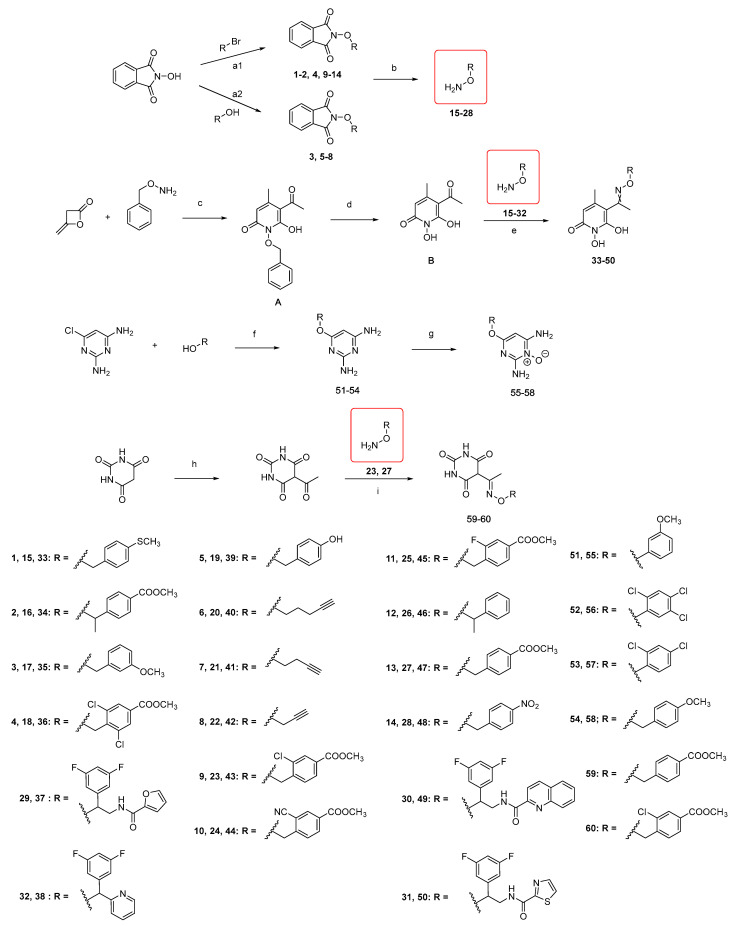
Synthesis of the *N*-hydroxypyridinediones 33–50, minoxidil analogs **55**–**58**, and barbituric acid analogs **59**–**60**. Reagents and conditions: (a1) NaH, DMF, 0 °C to RT, overnight; (a2) PPh3, DIAD, dry THF, Ar, 0 °C to RT, overnight–4 days (b) H_2_NNH_2_, DCM, or MeOH, RT, 1–16 h; (c) Et_3_N, dry toluene, 65 °C, 4 h; (d) H_2_, Pd/C 10%, RT, MeOH, 30 min; (e) EtOH, RT, overnight; (f) NaH 60% *w*/*w*, neat, 150–180 °C, 3 h–overnight; (g) mCPBA, MeOH, 0 °C, 3 h–overnight; (h) (Ac)_2_O, H_2_SO_4_, 110 °C (reflux), 1.5 h; (i) EtOH, RT, molecular sieves, reflux, 3 days.

The novel HPD compounds were synthesized using a three-step synthetic approach (Scheme 1). The first step involved the Gabriel reaction of *N*-hydroxyphthalimide with benzyl bromides or chlorides to afford the compounds **1**–**2**, **4**, and **9**–**14**. The *N*-hydroxyphthalimides **3** and **5**–**8** were synthesized using the Mitsunobu reaction, where triphenylphosphine reacts with diisopropyl azodicarboxylate (DIAD) to form a phosphonium intermediate. This intermediate then binds to the oxygen of the alcohol, facilitating a subsequent nucleophilic substitution. Subsequently, the *O*-substituted *N*-hydroxyphthalimides **1**–**14** reacted with hydrazine monohydrate to afford the corresponding *O*-substituted hydroxylamines **15**–**28**. In the final step, the suitable hydroxylamines were condensed with 5-acetyl-1-(benzyloxy)-6-hydroxy-4-methylpyridin-2(1*H*)-one **B** in absolute ethanol at reflux to yield the HPD oximes **33**–**50**. Oximes exhibit two geometrical isomers, E and Z. In the present work, no attempt was made to separate the two isomers of the final oxime derivatives and thus the compounds were obtained as a mixture, with a different ratio of the two isomers each time. The existence of the two isomers was confirmed by one- and two-dimensional NMR experiments and the E/Z (or Z/E) isomer ratio was calculated.

The key intermediate 5-acetyl-1-(benzyloxy)-6-hydroxy-4-methylpyridin-2(1*H*)-one **A** was synthesized, as we have previously reported, with an improved yield of 75%, compared with that of literature, by refluxing a mixture of diketene (2 equiv.) and *O*-benzyl hydroxylamine (1 equiv.) in the presence of triethylamine (1 equiv.) in anhydrous toluene. Afterwards, the catalytic hydrogenolysis of the benzyl group over 10% palladium on carbon yielded the target compound **B** almost quantitatively (Scheme 1) [30].

The analogs of minoxidil **55**–**58** were synthesized using a two-step synthetic approach (Scheme 1). The first step is a nucleophilic aromatic substitution of phenols or benzyl alcohols with 6-chloro-2,4-diaminopyrimidine and NaH base. Subsequently, the 6-substituted-2,4-diaminopyrimidines **51**–**54** are oxidized with mCPBA to afford the final minoxidil analogs **55**–**58**.

Analogs **59**–**60** of barbituric acid were prepared via a two-step reaction process originating from barbituric acid (Scheme 1). Initially, barbituric acid underwent acetylation using acetic anhydride under reflux. Subsequently, 5-acetyl barbituric acid was combined with *O*-substituted hydroxylamines (which were prepared as previously outlined) through a coupling reaction in absolute ethanol in the presence of molecular sieves, resulting in the formation of the compounds **59**–**60**.

**Scheme 2:**
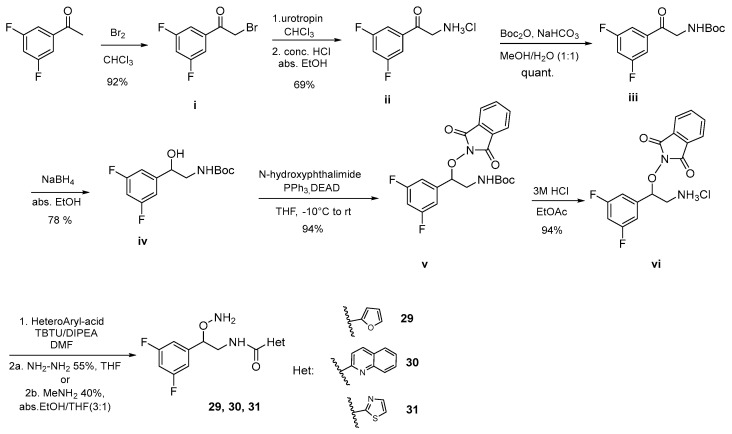
Synthesis of hydroxylamines **29**–**31.** Reagents and conditions: (a) Br_2_, CHCl_3_, RT, 2 h, 92%; (b) urotropine, CHCl_3_, RT, 4 h, then HCl 37%, EtOH, RT, overnight, 69%; (c) Boc_2_O, NaHCO_3_, MeOH/H_2_O (1:1), RT, 90 min, quant.; (d) NaBH_4_, EtOH, 0 °C, 1 h, 78%; (e) *N*-hydroxyphthalimide, DEAD, triphenylphosphine, THF, −10 °C to RT, overnight, 94%; (f) 3M HCl in AcOEt, RT, 90 min, 94%; (g) heteroarylacid, TBTU, DIPEA, DMF, RT, overnight, then aq. NH_2_NH_2_ (55%) THF, RT, 60–90 min, 67–85%, or aq. MeNH_2_ (40%), EtOH/THF 3:1, RT, overnight, 92%.

For the synthesis of the hydroxylamines **29**–**31**, a different synthetic procedure was employed (Scheme 2). The a-bromination of commercially available 3,5-difluoroacetophenone yielded the compound **i** in 92% yield. NMR analysis revealed the presence of a small quantity of unreacted starting material which was indistinguishable from the product during TLC monitoring of the reaction. Nevertheless, the purity of the crude product was acceptable (94% *w*/*w* by NMR analysis), and the impurity would be removed in the subsequent step. The application of the Delepine reaction yielded the hydrochloric salt of the amine **ii** in 69% yield after recrystallization. The amine was Boc-protected with Boc_2_O/NaHCO_3_ in a quantitative yield to afford the intermediate **iii** which was reduced to the corresponding alcohol **iv** with NaBH_4_ in a satisfactory yield of 78%. The hydroxylamine precursor moiety *N*-hydroxyphthalimide was then tethered to the compound **iv** under Mitsunobu reaction conditions to afford the intermediate **v** in an excellent yield (94%). Subsequently, the Boc group was cleaved with 3M HCl in AcOEt and the free amine **vi** was coupled to the corresponding heteroaryl acid with TBTU/DIPEA. After workup, the crude amide was subjected to hydrazinolysis or aminolysis with methylamine to afford the final hydroxylamine in good yields (67–92%) over two steps. 

The hydroxylamine **32** [*O*-(2-(3,5-difluorophenyl)-2-(pyridin-2-yl)ethyl)hydroxylamine], used for the synthesis of compound **38**, was synthesized by adopting a published synthetic methodology [33] for similar compounds and will be reported elsewhere.

### 2.2. Efficacy against HBV Replication and Cytotoxicity

Starting from the most promising HBV RNase H inhibitor we previously reported, A24 [29], we designed and synthesized 18 novel HPD oximes that bear the same pharmacophore scaffold as the hit compound and alterations in the side oxime moiety. Our goal was to further explore the SARs of the HPD scaffold to improve the compounds’ potency, selectivity, and druglike properties.

All 18 novel HPDs had EC_50_ values in the low μΜ range (1.1–7.7 μΜ). Moreover, 15 out of 18 exhibited no significant cytotoxicity in vitro (CC_50_ values > 80 μΜ), resulting in SI values (CC_50_/EC_50_) ranging from 11.9 to 91.7 (Table 1).

The compounds **47** and **48** were the most potent among the newly synthesized compounds (EC_50_ of 1.1 μΜ). Both compounds feature the same benzyl group side chain as A24 but differ in their 4′-substitution on the side benzene ring; **47** bears a 4′-COOCH_3_ group, while **48** hosts a 4′-NO_2_ group. Notably, all three compounds share a polar group at the 4′-position of the side benzene, indicating the potential favorability of incorporating polar groups in this position for enhanced antiviral activity. Moreover, **47** and **48** are minimally cytotoxic, yielding SI values of 87.9 and 91.7, respectively.

The compounds **34** and **46** are characterized by a methyl group positioned on the benzylic methylene, linking the core ring to the side aromatic moiety. This linker group appears pivotal in the inhibitory activity, as evidenced by an up to sixfold reduction in antiviral efficacy (EC_50_ = 6.3 μΜ for **34**) compared to the structurally analogous compound **47** that also features a 4′-COOCH_3_ substitution.

We also investigated the impact of increasing the size of the side aromatic moiety by incorporating additional aromatic and heteroaromatic rings (the compounds **37**, **38**, **49**, and **50**). Remarkably, these compounds were active against HBV replication, with EC_50_ values ranging from 2.3 to 4.7 μM. Moreover, three out of the four compounds with the bulkier side moiety exhibited no cytotoxic effects, thereby maintaining favorable SI values.

Analogs with a single substitution on the side chain (**33**–**35**, **39**–**42**, **47**–**48**) demonstrate greater potency compared to those with two or three substitutions (**36**–**38**, **43**–**45**, **49**, **50**), regardless of the nature of the substitution. Notably, compounds containing halogens exhibited a significant increase in inhibitory activity, particularly when the halogen is positioned at the 4′ location of the aromatic side chain (**33**, **34**, **47**, **48**). This suggests that non-polar groups enhance potency and reduce cytotoxicity more effectively than polar groups, such as the hydroxyl group in the compound **39**. This is likely due to the hydrophobic loop in the enzyme’s active site, which necessitates a lipophilic moiety in the compound, thereby enhancing ligand–protein interactions.

We also tested the significance of the aromaticity by incorporating aliphatic side chains (**40**, **41**, **42**). The results showed the favorability of the four-carbon chain with an EC_50_ 1.2 μΜ for the compound **41** and good SI values.

Overall, HPDs featuring aromatic side chains substituted at the 4′ position with non-polar groups were the most effective HBV RNase H inhibitors. Conversely, larger polar groups, particularly two or three aromatic rings, as well as the inclusion of a chiral linker, are less well tolerated for antiviral efficacy. Compounds that possess halogen substitutions on the aromatic side chain and a short linker (1 C after the oxime group) between the HPD core pharmacophore ring and the side aromatic moiety exhibit the optimal combination of antiviral activity, minimal cytotoxicity, and favorable SI values.

Our next effort focused on structurally modifying the primary pharmacophore by incorporating the minoxidil structure. Given the structural similarity between minoxidil and our HPD pharmacophore, we hypothesized that the three electron donors (two N atoms and one O atom) would form a “trident” configuration capable of chelating the Mg^2+^ ions at the enzyme’s catalytic site. Although computational studies (see Section 2.4) suggested potential activity, the compounds **55**–**58** were inactive against viral replication in vitro. Several hypotheses could explain these results, including the reduced electronegativity of nitrogen compared to oxygen (existent in the HPD heteroatom “trident”), the compounds’ potential inability to penetrate cell membranes due to increased polarity, or the absence of E/Z isomerism, which might influence the molecule’s conformation within the catalytic site. Further optimization could shed light on the potential antiviral activity of these analogs.

Despite their ability to chelate the Mg^2+^ ions in the enzyme catalytic site in computational studies (see Section 2.4), the compounds **59** and **60**, where the HPD ring was replaced with the pharmacophore ring of barbituric acid, lacked any inhibitory activity. This may be because the oxygen “trident” of the *N*-hydroxyimide group is essential for coordinating metal ions within the RNase H active site. These findings align with our previous observations for this class of compounds [30]. Additionally, the increased polarity of these compounds likely reduces their inability to cross cell membranes to come into contact with the enzyme. To further address the impact of the different substitution patterns and residues in lipophilicity and druggability, we conducted a modeling calculation of druglike properties and descriptors using QikProp module of the Schrödinger platform (Table S1, Supporting Information).

### 2.3. Compound Solubility and Apparent Passive Permeability

In total, 21 out of the 24 compounds were highly soluble (solubility limit ≥ 100 μM) in conditions reflecting tissue culture media (pH 7.4). However, the barbituric analogs were insoluble, preventing meaningful biological evaluation (Table 2). In summary, the HPD oximes and minoxidil analogs exhibit promising druglike properties (Table 1 and Table S1). Additionally, out of the 24 novel compounds tested in parallel artificial membrane assays, 22 demonstrated a high apparent passive permeability by the industry standard cutoff (>1 × 10^−6^ cm/s). This includes all HPD oximes and minoxidil analogs.

### 2.4. Computational Molecular Docking

**Figure 3:**
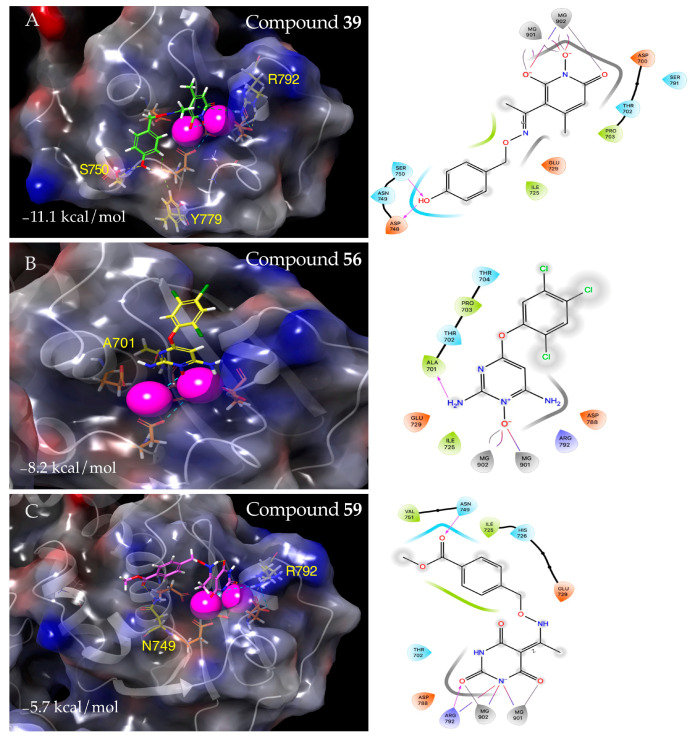
HPDs chelating Mg^2+^ ions. (**A**) The compound **39** (green, pKa = 9.609) docked into the active site of HBV P RNase H domain chelating both Mg^2+^ ions. The ligand interaction diagram on the right shows two deprotonated hydroxyl groups on the HPD core making 7 salt bridge bonds (**--**) and 1 metal coordination interaction (**--**). In (**B**,**C**), the compounds **56** (yellow, pKa = 7.509) and **59** (pink) make 3 salt bridge (**--**) interactions with Mg^2+^ ions. **Right**; ligand interaction diagram; **Left**, surface diagram. A PDB file containing for the HBV RNase H used for docking can be found in [18]. Docking scores are in kCal/mol.

We performed induced fit docking (IFD) experiments to evaluate the binding pattern for all compounds into the active site of RNase H domain of HBV P. IFD was restricted to produce at least five binding poses for each compound. All of the compounds chelated two Mg^2+^ ions via salt bridge interactions. It was observed in the case of the co-crystal structure of HIV RNase H with β-thujaplicinol that the hydroxyl trident of the inhibitor chelates Mg^2+^ ions via eight salt bridge bonds. The core of the HPDs have two adjacent hydroxyl groups able to chelate both Mg^2+^ ions via 6–7 salt bridge interactions, while minoxidil derivatives (**55**–**58**) containing only one hydroxyl group can only make 2 to 4 bonds (Figure 3A,B). The compounds **59** and **60** which are analogs to barbituric acid contain an alternative carbonyl on their main core and can chelate Mg^2+^ ions via their deprotonated amino group through 1 to 2 salt bridge interactions (Figure 3C). The reduced number of interactions with Mg^2+^ ions are also reflected in their poor docking scores −5.7 and −5.5 kcal/mol for **59** and **60**, respectively (Table 1). We also observed that the compounds **56**, **57**, and **58** which form 4 or fewer salt bridge interactions have EC_50_ values >100 μM as docking scores range from −8.2 to −8.17 kcal/mol, whereas compounds which contain at least two hydroxyl groups on their main core have docking scores ranging from −11.1 to −8.9 kcal/mol and have EC_50_ values ranging from 1.1 to 7.7 μM.

**Figure 4:**
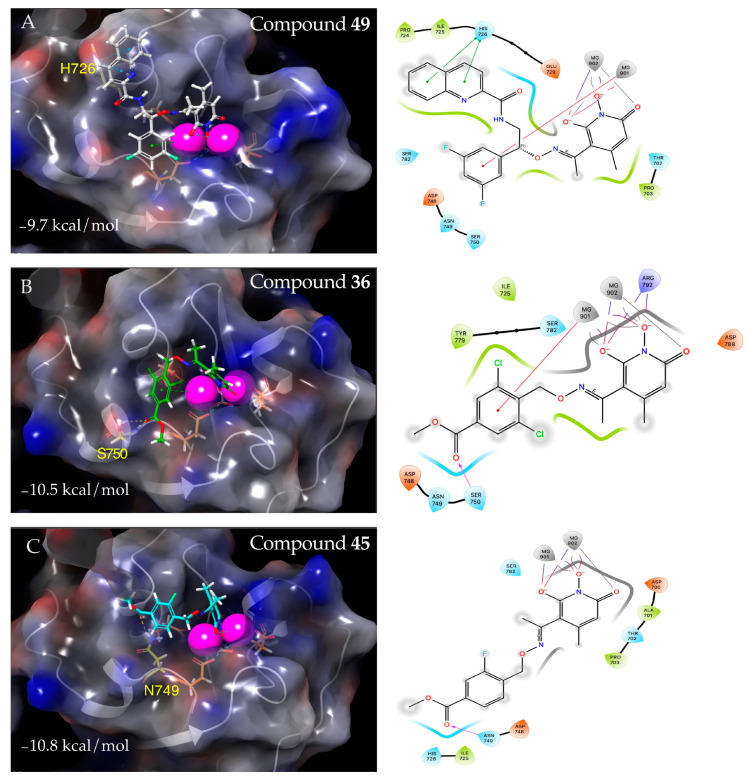
Docking studies of HPDs into the active site of RNase H domain of HBV P. (**A**) The R-group of the compound **49** (white) docked into the active site of the HBV P RNase H domain makes a pi–pi cation interaction with H726 in the S3 binding pocket. (**B**) The compound **36** (green) R-group makes an h-bond with S750, whereas (**C**) **45** (cyan) makes an h-bond with N749 in the S3 binding pocket. **Right panel**, ligand interaction map; **Left panel**, surface diagram of docked compound into the active site of HBV P RNase H domain. A PDB file containing for the HBV RNase H used for docking can be found in [18]. Docking scores are in kCal/mol.

In our previous docking results, the R-groups of most of the compounds were solvent exposed and few of them interacted in the three binding pockets which were defined based on the R-group placement [30]. In our current docking study, most of the compounds’ R-groups were located in pocket S3 and made interactions with residues S750, N749, and H726 in the pocket (Figure 4). It seems that a longer oxime linker helps these compounds to fit better into the S3 binding pocket to facilitate the formation of interactions with residues in the binding pocket.

## 3. Materials and Methods

### 3.1. Chemistry—General Part

All reagents and starting materials were purchased from commercial suppliers and used without further purification. Anhydrous CH_2_Cl_2_ was obtained by distillation from calcium hydride under argon. Anhydrous THF was freshly distilled from Na and benzophenone ketyl. All non-aqueous reactions were performed under an inert atmosphere of argon. Concentrated refers to the removal of solvent with a rotary evaporator at normal water aspirator pressure, followed by further evacuation on a high-vacuum line. Thin-layer chromatography was performed using silica gel 60 Å precoated aluminum or glass-backed plates (0.25 mm thickness) with fluorescent indicators. Developed TLC plates were visualized with UV light (254 nm), iodine vapors, or anisaldehyde staining solution. The chromatographic purification of the products was carried out using Fluka silica gel 60 (Honeywell, Morris Plains, NJ, USA) for preparative column chromatography (particle size 40–63 μm). Melting points were determined using a Büchi 530 device (Flawil, Switzerland) and presented without using corrections. NMR spectra were obtained in CDCl_3_ or DMSO-*d*_6_ at 25 °C on a Bruker Avance DRX 600, 500, 400, or 250 MHz spectrometer. The measured chemical shifts are reported in δ (ppm), and the residual solvent signal was used as the internal calibration standard (CDCl_3_): ^1^H = 7.26 ppm, ^13^C = 77.18 ppm); (DMSO-*d*_6_): ^1^H = 2.50 ppm, ^13^C = 39.51 ppm. ^13^C-NMR spectra were obtained with complete proton decoupling. The data of NMR spectra were recorded as follows: s = singlet, d = doublet, t = triplet, m = multiplet, dd = doublet of doublets, td = triplet of doublets, tt = triplet of triplets, and br = broad signal. The coupling constant *J* is reported in hertz (Hz). ^1^H- and ^13^C-NMR peaks were assigned based on the combined analysis of a series of ^1^H-^1^H (COSY) and ^1^H-^13^C (HSQC, HMBC) correlation spectra.

### 3.2. Chemistry—Experimental Procedures and Compound Characterization

#### 3.2.1. Synthesis of 5-Acetyl-1,6-dihydroxy-4-methylpyridin-2(1*H*)-one (**Β**)

To produce 5-Acetyl-1-(benzyloxy)-6-hydroxy-4-methylpyridin-2(1H)-one (**A**), a stirred solution of *O*-benzylhydroxylamine (2.98 g, 24.2 mmol, 1.0 equiv.) and triethylamine (2.45 g, 3.38 mL, 24.2 mmol, 1.0 equiv.) in 19 mL dry toluene was cooled in an ice-bath, and diketene (4.07 g, 3.73 mL, 48.4 mmol, 2.0 equiv.) was added dropwise. After 4.5 h of stirring at 65 °C under argon, the mixture was concentrated to dryness under reduced pressure and treated with 150 mL HCl 10%. The residue was partitioned between the aqueous phase and AcOEt (300 mL), the organic phase was extracted once more with HCl 10% (150 mL), and the combined aqueous phases were extracted once more with 150 mL AcOEt. The combined organic phases were washed with brine (3 × 200 mL), dried over anhydrous Na_2_SO_4_, and the solvent was removed in vacuo. The residual brownish solid was triturated with Et_2_O and AcOEt sequentially to afford the title compound A as a beige crystalline solid (4.95 g, 75%); mp 144–146 °C (MeOH, AcOEt/*n*-pentane), R*_f_* _(*NP-TLC*)_ = 0.25 (AcOEt). ^1^H NMR (600.11 MHz, DMSO-*d*_6_) δ 2.34 (s, 3H, 4-C*H*_3_), 2.60 (s, 3H, 7-C*H*_3_), 5.05 (s, 2H, C*H*_2_Ph) 5.83 (s, 1H, *H*_3_), 7.37–7.44 (complex m, 3H, H_3′_, H_4′_, H_5′_), 7.54 (dd, 2H, *J*_1_ = 7.5 Hz, *J*_2_ = 1.7 Hz, H_2′_, H_6′_). ^13^C NMR (50.32 MHz, DMSO-*d*_6_) δ 23.8 (4-*C*H_3_), 29.0 (7-*C*H_3_), 76.9 (*C*H_2_Ph), 104.8 (C_5_), 106.7 (C_3_), 128.2 (C_3′_, C_5′_), 128.6 (C_4′_), 129.3 (C_2′_, C_6′_), 134.9 (C_1′_), 150.5 (C_4_), 159.0 (C_2_), 164.8 (C_6_), 193.4 (C_7_). Elemental analysis calcd (%) for C_15_H_15_NO_4_: C, 65.92; H, 5.53; N, 5.13; found: C, 66.00; H, 5.59; N, 5.08 [30].

To produce 5-Acetyl-1,6-dihydroxy-4-methylpyridin-2(1*H*)-one (**B**), a solution of **A** (2.0 g, 7.32 mmol) in 120 mL MeOH was hydrogenated for 20 min at rt and 40 psi pressure, in the presence of 200 mg Pd/C (10 wt.%) as a catalyst. The catalyst was filtered off, washed with portions of hot MeOH (3 × 20 mL) and the combined filtrates were evaporated under reduced pressure. The beige crystalline product was treated with AcOEt to yield the *N*-hydroxypyridinedione **B** almost quantitatively (1.32 g, 98%); m.p. 178–179 °C (MeOH/*n*-pentane, dry Et_2_O), R*_f_*_(*NP-TLC*)_ = 0.06 (AcOEt), R*_f_*_(*RP-TLC*)_ = 0.85 (H_2_O/ACN 7:3). ^1^H NMR (600.11 MHz, DMSO-*d*_6_) δ 2.32 (s, 3H, 4-C*H*_3_), 2.56 (s, 3H, 7-C*H*_3_), 5.80 (s, 1H, H_3_). ^13^C NMR (100.61 MHz, DMSO-*d*_6_) δ 23.3 (4-*C*H_3_), 28.0 (7-*C*H_3_), 104.7 (C_5_), 107.6 (C_3_), 149.1 (C_4_), 157.9 (C_2_), 165.1 (C_6_), 194.3 (C_7_). Elemental analysis calcd (%) for C_8_H_9_NO_4_: C, 52.46; H, 4.95; N, 7.65; found: C, 52.51; H, 5.00; N, 7.58 [30].

#### 3.2.2. Synthesis of *O*-Substituted *N*-Hydroxyphthalimides **1**–**14**

General procedure:

To a solution of *N*-hydroxyphthalimide (500.0 mg, 3.07 mmol, 1 equiv.) in anhydrous DMF (3 mL), NaH 60% *w*/*w* (1.25 equiv.) is added at 0 °C. The mixture is stirred at rt for 30 min. Thereafter, the appropriate halogenide (1.5 equiv.) is added and the reaction is stirred at rt overnight. Then, water is added and a solid precipitate is formed. The precipitate is filtered under vacuum and washed with water and a solution of *n*-pentane/Et_2_O 7:3. The solid is dried over P_2_O_5_, to afford the desired product.

The compound 2-(4-(Methylthio)benzyl-oxy)isoindolin-1,3-dione (**1**) was synthesized from (4-(bromomethyl)phenyl)(methyl)sulfane according to the general procedure. White solid (697.7 mg, 95%). R*_f_* = 0.54 (CH_2_Cl_2_), m.p. 147 °C, ^1^H NMR (500 MHz, CDCl_3_) δ 7.81 (dd, *J* = 5.5, 3.1 Hz, 2H, Ar-Pthal), 7.73 (dd, *J* = 5.5, 3.1 Hz, 2H, Ar-Pthal), 7.45 (d, *J* = 8.3 Hz, 2H, Ar), 7.24 (d, *J* = 8.3 Hz, 2H, Ar), 5.17 (s, 2H, OCH_2_), 2.48 (s, 3H, SCH_3_). ^13^C NMR (126 MHz, CDCl_3_) δ 163.67 (C_1_, C_3_), 140.40 (C_4′_), 134.59 (C_2′_), 130.56 (C_7a_, C_3a_), 130.31 (C_6_, C_5_), 128.98 (C_5′_, C_3′_), 126.21 (C_6′_, C_2′_), 123.67 (C_7_, C_4_), 79.54 (1′-CH_2_), 15.55 (S-CH_3_). Elemental analysis calcd (%) for C_16_H_13_NO_3_S: C, 64.20; H, 4.38; N, 4.68. Found: C, 64.24; H, 4.42; N, 4.72.

The compound Methyl 4-[1-(1,3-dioxoisoindole-2-yl-oxy)ethyl] benzoate (**2**) was synthesized from methyl 4-(1-bromoethyl)benzoate (1.5 equiv.) according to the general procedure. White solid (136.7 mg, 74%). R*_f_* = 0.35 (CH_2_Cl_2_), m.p. 170–171 °C, ^1^H NMR (600 MHz, CDCl3) δ 8.01 (d, *J* = 8.3 Hz, 2H, Ar), 7.75 (dd, *J* = 5.4, 3.1 Hz, 2H, Ar-Pthalimide), 7.69 (dd, *J* = 5.5, 3.1 Hz, 2H, Ar-Pthalimide), 7.63–7.57 (m, 2H, Ar), 5.54 (q, *J* = 6.5 Hz, 1H, CH-CH_3_), 3.90 (s, 3H, CH_3_), 1.72 (d, *J* = 6.5 Hz, 3H, CH-CH_3_). ^13^C NMR (151 MHz, CDCl_3_) δ 166.65 (COOCH_3_), 163.66 (C_1_, C_3_), 144.14 (C_4′_), 134.35 (C_2′_, C_6′_), 130.59 (C_7a_, C_3a_), 129.65 (C_6_, C_5_), 128.76 (C_1′_), 127.42 (C_3′_, C_5′_), 123.41 (C_7_, C_4_), 84.54 (C_8_), 52.04 (COOCH_3_), 20.62 (8-CH_3_). Elemental analysis calcd (%) for C_18_H_15_NO_5_: C, 66.46; H, 4.65; N, 4.31. Found: C, 66.49; H, 4.69; N, 4.35.

The compound 2-(3-Methoxybenzyloxy)isoindolin-1,3-dione (**3**) was synthesized as follows: To a solution of *N*-hydroxyphthalimide (200 mg, 1.23 mmol, 1 equiv.) in dry THF (10 mL), 1-(bromomethyl)-3-methoxybenzene (1.84 mmol, 1.5 equiv.) and PPh_3_ (353.7 mg, 1.35 mmol, 1.1 equiv.) are added. Then, DIAD (265 μL, 1.35 mmol, 1.1 equiv.) is added dropwise at 0 °C. The reaction is stirred at rt for 48–70 h. The reaction mixture is extracted from EtOAc (3 × 50 mL) and the combined organic phases are washed with brine, dried over anh. Na_2_SO_4_, filtered, and concentrated. The resulting crude mixture is purified by recrystallization (EtOH). White foamy solid (214.6 mg, 62%). R*_f_* = 0.5 (AcOEt), m.p. 120–121 °C. ^1^H NMR (600 MHz, CDCl_3_) δ 7.81 (dd, *J* = 5.4, 3.1 Hz, 2H, Ar-Pthalimide), 7.73 (dd, *J* = 5.5, 3.1 Hz, 2H, Ar-Pthalimide), 7.31–7.26 (m, 1H, Ar), 7.14–7.06 (m, 2H, Ar), 6.91 (ddd, *J* = 8.3, 2.6, 1.0 Hz, 1H, Ar), 5.20 (s, 2H, OCH_2_), 3.83 (s, 3H, OCH_3_). ^13^C NMR (126 MHz, CDCl_3_) δ 163.87 (C_1_, C_3_), 160.08 (C_3′_), 135.58 (C_1′_), 134.82 (C_5′_), 129.94 (C_7a_, C_3a_), 129.30 (C_6_, C_5_), 123.89 (C_6′_), 122.34 (C_7_, C_4_), 115.82 (C_4′_), 115.02 (C_2′_), 80.13 (1′-CH_2_), 55.70 (O*CH3*). Elemental analysis calcd (%) for C_16_H_13_NO_4_: C, 67.84; H, 4.63; N, 4.94. Found: C, 67.88; H, 4.67; N, 4.98 [35].

The compound Methyl 3,5-dichloro-4-(((1,3-dioxoisoindolin-2-yl)oxy)methyl)benzoate (**4**) was synthesized from methyl 4-(bromomethyl)-3,5-dichlorobenzoate (300.0 mg, 1.01 mmol) according to the general procedure. Pink solid (226.9 mg, 89%). R*_f_* = 0.67 (CH_2_Cl_2_), m.p. 145–147 °C. ^1^H NMR (400 MHz, CDCl_3_) δ 8.09–7.89 (m, 4H, Phth), 7.87 (s, 2H, Ar), 4.79 (s, 2H, CH_2_), 3.89 (s, 3H. CH_3_) ppm. ^13^C NMR (125 MHz, CDCl_3_) δ 164.33, 163.26, 133.90, 133.84, 133.19, 129.52, 128.63, 128.10, 123.31, 77.51, 52.41 ppm. Elemental analysis calcd (%) for C_17_H_11_Cl_2_NO_5_: C, 53.71; H, 2.92; N, 3.68; found: C, 53.69; H, 2.93; N, 3.68.

The compound 2-((4-Hydroxybenzyl)oxy)isoindoline-1,3-dione (**5**) was synthesized from 4-(bromomethyl)phenol (1 mmol) according to the procedure followed for the compound 3 (52 h). The compound underwent purification with column chromatography (1:1 AcOEt: Hexane, 2:1 AcOEt:Hexane, AcOEt) and recrystallization from EtOH. White solid (225 mg, 30%), R*_f_* = 0.28 (AcOEt), mp: 145–148 °C. ^1^H NMR (600 MHz, DMSO) δ 9.59 (s, 1H, OH), 7.85 (s, 4H, Ar-Pthalimide), 7.31–7.25 (m, 2H, Ar), 6.76 (d, *J* = 8.5 Hz, 2H, Ar), 5.03 (s, 2H, OCH_2_). ^13^C NMR (151 MHz, DMSO) δ 163.15 (C=O), 158.19 (C_4′_), 134.76 (C_3a_, C_7a_, C_6_, C_5_), 131.58 (C_2′_, C_6′_), 128.48 (C_1′_), 124.34, 123.18 (C_4_, C_7_), 115.16 (C_3′_, C_5′_), 79.06 (OCH_2_). Elemental analysis calcd (% for C_15_H_11_NO_4_: C, 66.91; H, 4.12; N, 5.20. Found: C, 66.95; H, 4.15; N, 5.24 [36].

The compound 2-(Pent-4-yn-1-yloxy)isoindoline-1,3-dione (**6**) was synthesized from pentil-1-ol (1 equiv.) according to the procedure followed for the compound 3 (48 h). The crude yellow solid was purified with column chromatography (7:3 HexaneL AcOEt) and recrystallized from EtOH. White solid (528 mg, 64%). R*_f_* = 0.5 (AcOEt), m.p. 80–82 °C, ^1^H NMR (600 MHz, DMSO) δ 7.86 (s, 4H, Ar), 4.21 (t, *J* = 6.3 Hz, 2H, OCH_2_), 2.79 (t, *J* = 2.7 Hz, 1H, C≡CH), 2.39 (td, *J* = 7.1, 2.7 Hz, 2H, CH_2_C≡CH), 1.88–1.82 (m, 2H, CH_2_C*H*_2_CH_2_), (100 MHz, CDCl_3_) δ 163.63 (C=O), 134.52 (C_3a_, C_7a_, C_6_, C_5_), 128.92, 123.55 (C_4_, C_7_), 83.08 (C_4′_), 76.91 (C_5′_), 69.11 (C_1′_), 27.21 (C_2′_), 14.94 (C_3′_). Elemental analysis calcd (%) for C_13_H_11_NO_3_: C, 68.11; H, 4.84; N, 6.11. Found: C, 68.15; H, 4.88; N, 6.15 [37].

The compound 2-(But-3-yn-1-yloxy)isoindoline-1,3-dione (**7**) was synthesized from butin-1-ol (1 equiv.) according to the procedure followed for compound **3** (48 h). The compound underwent purification by column chromatography (7: 3 Hexane: AcOEt) and recrystallization from EtOH. White solid (473 mg, 60%). R*_f_* = 0.45 (AcOEt), m.p. 104 °C. ^1^H NMR (600 MHz, CDCl_3_) δ 7.85 (dd, *J* = 5.4, 3.1 Hz, 2H, Ar-Pthalimide), 7.76 (dd, *J* = 5.4, 3.1 Hz, 2H, Ar), 4.33 (t, *J* = 7.1 Hz, 2H, OCH_2_), 2.74 (d, *J* = 2.7 Hz, 2H, OCH_2_C*H_2_*), 1.98 (s, 1H, C≡CH). ^13^C NMR (125 MHz, CDCl_3_) δ 163.6 (C=O), 134.7 (C_3a_, C_7a_, C_6_, C_5_), 128.9, 123.7 (C_4_, C_7_), 79.2 (C_3′_), 75.6 (C_4′_), 70.4 (C_1′_), 18.8 (C_2′_). Elemental analysis calcd (%) for C_12_H_9_NO_3_: C, 66.97; H, 4.22; N, 6.51. Found: C, 67.01; H, 4.26; N, 6.55 [38].

The compound 2-(Prop-2-yn-1-yloxy)isoindoline-1,3-dione (**8**) was synthesized from propin-1-ol (1 equiv.) according to the procedure followed for the compound 3 (48 h). The compound underwent purification by column chromatography (7: 3 Hexane: AcOEt) and recrystallization from EtOH. White solid (389.1 mg, 53%). R*_f_* = 0.33 (AcOEt), m.p. 149–150 °C. ^1^H NMR (500 MHz, CDCl_3_) δ 7.82 (ddd, *J* = 45.7, 5.5, 3.1 Hz, 4H, Ar), 4.88 (d, *J* = 2.4 Hz, 2H, OCH_2_), 2.59 (t, *J* = 2.4 Hz, 1H, C≡CH). ^13^C NMR (126 MHz, CDCl_3_) δ 163.49 (C=O), 134.78 (C_3a_, C_7a_, C_6_, C_5_), 128.88, 123.86 (C_4_, C_7_), 78.29 (C_2′_), 76.50 (C_3′_), 65.13 (C_1′_). Elemental analysis calcd (%) for C_11_H_7_NO_3_: C, 65.67; H, 3.51; N, 6.96. Found: C, 65.70; H, 3.54; N, 6.99 [39].

The compound Methyl 4-(((1,3-dioxoisoindolin-2-yl)oxy)methyl)-6′-chlorobenzoate (**9**) was synthesized from methyl 4-(bromomethyl)-3-chlorobenzoate (1 equiv.), according to the general procedure. White solid (150.5 mg, 86%). R*_f_* = 0.53 (CH_2_Cl_2_), m.p. 165–168 °C. ^1^H NMR (600 MHz, CDCl_3_) δ 8.06 (d, *J* = 1.6 Hz, 1H, H_3′_), 7.97 (dd, *J* = 8.0, 1.7 Hz, 1H, H_5′_), 7.83 (dd, *J* = 5.4, 3.1 Hz, 2H, Ar-Phth), 7.78 (dd, *J* = 8.0, 0.5 Hz, 1H, H_6′_), 7.75 (dd, *J* = 5.5, 3.0 Hz, 2H, Ar-Phth), 5.40 (s, 2H, OCH_2_), 3.93 (s, 3H, OCH_3_). ^13^C NMR (100 MHz, DMSO) δ 165.9 (C-O), 161.0 (C_1_, C_3_), 142.5 (C_1′_), 133.4 (C_4′_), 132.2 (C_2′_), 132.2 (C_5_, C_6_), 132.0 (C_3a_, C_7a_), 130.1 (C_3′_), 128.4 (C_6′_), 128.2 (C_5′_), 123.7 (C_4_, C_7_), 68.5 (OCH_2_), 51.5 (OCH_3_). Elemental analysis calcd (%) for C_17_H_12_ClNO_5_: C, 59.06; N, 4.05; H, 3.50. Found: C, 59.09; N, 4.09; H, 3.54.

The compound Methyl 4-(((1,3-dioxoisoindolin-2-yl)oxy)methyl)-6′-cyanobenzoate (**10**) was synthesized from methyl 4-(bromomethyl)-3-cyanobenzoate (1equiv.) according to the general procedure. White solid (102.7 mg, 89.5%). R*_f_* = 0.53 (CH_2_Cl_2_), m.p. 165–168 °C. ^1^H NMR (600 MHz, CDCl_3_) δ 8.34 (d, *J* = 1.7 Hz, 1H, Ar), 8.30 (dd, *J* = 8.1, 1.8 Hz, 1H, Ar), 7.94 (dd, *J* = 8.2, 0.6 Hz, 1H, Ar), 7.85–7.74 (m, 4H, Ar-Phth), 5.47 (s, 2H, OCH_2_), 3.96 (s, 3H, OCH_3_). ^13^C NMR (100 MHz, CDCl_3_) δ 165.9 (C-O), 161.0 (C_1_, C_3_), 148.2 (C_1′_), 129.7 (C_4′_), 111.3 (C_2′_), 132.2 (C_5_, C_6_), 132.0 (C_3a_, C_7a_), 133.3 (C_3′_), 127.7 (C_6′_), 134.4 (C_5′_), 123.7 (C_4_, C_7_), 115.8 (C-N), 70.3 (OCH_2_), 51.5 (OCH_3_). Elemental analysis calcd (%) for C_18_H_12_N_2_O_5_: C, 64.29; N, 8.33; H, 3.60. Found: C, 64.31; N, 8.37; H, 3.61.

The compound Methyl 4-(((1,3-dioxoisoindolin-2-yl)oxy)methyl)-3-fluorobenzoate (**11**) was synthesized from methyl 4-(bromomethyl)-3-fluorobenzoate (1 equiv.) according to the general procedure. White solid (139.8 mg, 79%). R*_f_* = 0.12 (CH_2_Cl_2_), m.p. 165–168 °C. ^1^H NMR (600 MHz, CDCl_3_) δ 7.85 (dd, *J* = 7.9, 1.6 Hz, 1H, Ar), 7.81 (dd, *J* = 5.4, 3.1 Hz, 2H, Ar-Phth), 7.76–7.73 (m, 2H, Ar-Phth), 7.73–7.67 (m, 2H, Ar), 5.33 (d, *J* = 1.1 Hz, 2H, OCH_2_), 3.92 (s, 3H, CH_3_). ^13^C NMR (151 MHz, CDCl_3_) δ 165.51 (COOCH_3_), 163.21 (C_1_, C_3_), 160.14 (C_3′_), 134.51 (C_7a_, C_3a_), 133.21 (C_6_, C_5_), 133.15 (C_4′_), 131.74 (C_1′_), 126.23 (C_5′_), 125.34 (C_6′_), 123.58 (C_7_, C_4_), 116.66 (C_2′_), 72.47 (4′-CH_2_), 52.38 (COO*CH_3_*). Elemental analysis calcd (%) for C_17_H_12_FNO_5_: C, 62.01; H, 3.67; N, 4.25. Found: C, 62.05; H, 3.71; N, 4.29.

The compound 2-(1-Phenylethoxy)isoindoline-1,3-dione (**12**) was synthesized as follows. *N*-hydroxyphthalimide (500.0 mg, 3.07 mmol, 1 equiv.) was dissolved in DMSO (5.2 mL). Then, Na_2_CO_3_ (976.2 mg, 9.21 mmol, 3 equiv.) and (1-bromoethyl)benzene (1.70 g, 9.21 mmol, 3 equiv.) were added successively. The resulting mixture was stirred for 16 h at rt under argon. Subsequently, water (50 mL) was added, and the formed white precipitate was filtered, washed with water, dried for 24 h and recrystallized (EtOH) to afford the title compound as a white solid (305.0 mg, 37%). ^1^H NMR (600 MHz, CDCl_3_) δ 7.75 (dd, *J* = 5.4, 3.1 Hz, 2H), 7.69 (dd, *J* = 5.5, 3.0 Hz, 2H), 7.52–7.49 (m, 2H), 7.36–7.29 (m, 3H), 5.50 (q, *J* = 6.5 Hz, 1H), 1.72 (d, *J* = 6.5 Hz, 3H) ppm [40].

The compound Methyl 4-(((1,3-dioxoisoindolin-2-yl)oxy)methyl)benzoate (**13**) was synthesized from methyl 4-(bromomethyl)benzoate (1equiv.) according to the general procedure. Pink solid (758.3 mg, 99%). R*_f_* = 0.53 (CH_2_Cl_2_), m.p. 155–158 °C. ^1^H NMR (400 MHz, CDCl_3_) δ 8.08–8.02 (m, 2H, Ar-Phth), 7.85–7.79 (m, 2H, Ar-Phth), 7.76–7.72 (m, 2H, Ar), 7.65–7.59 (m, 2H, Ar), 5.26 (s, 2H, OCH_2_), 3.92 (s, 3H, OCH_3_). ^13^C NMR (151 MHz, CDCl_3_) δ 165.9 (C-O), 163.40 (C_1_, C_3_), 140.8 (C_1′_), 132.2 (C_5_, C_6_), 132.0 (C_3a_, C_7a_), 130.1 (C_3′_, C_5′_), 129.3 (C_2′_, C_6′_), 129.0 (C_4′_), 123.7 (C_4_, C_7_), 78.3 (CH_2_), 51.5 (CH_3_). Elemental analysis calcd (%) for C_15_H_10_ClNO_3_: C, 65.17; N, 4.50; H, 4.83. Found: C, 65.20; N, 4.53; H, 4.85 [41].

The compound 2-((4-Nitrobenzyl)oxy)isoindoline-1,3-dione (**14**) was synthesized from 1-(bromomethyl)-4-nitrobenzene (500.0 mg, 2.31 mmol) according to the general procedure. Yellow solid (423.9 mg, 92%). ^1^H NMR (400 MHz, CDCl_3_) δ 8.25 (d, *J* = 8.7 Hz, 2H), 7.86–7.76 (m, 4H), 7.74 (d, 2H), 5.31 (s, 2H) ppm [42].

#### 3.2.3. Synthesis of *O*-Substituted Hydroxylamines **15–28**

General procedure:

To a solution of the appropriate *N*-hydroxyphthalimide (250.0 mg, 1 equiv.) in CH_2_Cl_2_ (3 mL), hydrazine monohydrate 64% *w*/*w* (2 equiv.) is added and the reaction is stirred at rt for 1–24 h. The formed white precipitate is filtered, washed with CH_2_Cl_2_, and the filtrate is concentrated to afford the corresponding hydroxylamine.

The compound *O*-(4-(Methylthio)benzyl)hydroxylamine (**15**) was synthesized from the compound 1 (1 equiv.) according to the general procedure, to afford an off-yellow oil (154.8 mg, 97%). R*_f_* = 0.14 (CH_2_Cl_2_). ^1^H NMR (600 MHz, CDCl_3_) δ 7.28 (d, *J* = 8.5 Hz, 2H, Ar), 7.26–7.23 (m, 2H, Ar), 5.37 (s, 2H, NH_2_), 4.64 (s, 2H, OCH_2_), 2.48 (s, 3H, CH_3_). ^13^C NMR (125 MHz, CDCl_3_) δ 138.04 (C_4_), 133.21 (C_1_), 128.26, 127.65 (C_2_, C_3_, C_5_, C_6_), 77.76 (OCH_2_), 15.52 (SCH_3_).

The compound Methyl 4-(1-(aminooxy)ethyl)benzoate (**16**) was synthesized from the compound **2** (1 equiv.) according to the general procedure. Off-yellow oil (66.7 mg, 88%). R*_f_ =* 0.29 (CH_2_Cl_2_). ^1^H NMR (400 MHz, CDCl_3_) δ 8.07–7.98 (m, 2H, Ar), 7.41 (d, *J* = 8.0 Hz, 2H, Ar), 5.29 (s, 2H, NH_2_), 4.72 (d, *J* = 7.1 Hz, 1H, C*H*CH_3_), 4.02–3.84 (m, 3H, CH_3_). ^13^C NMR (126 MHz, CDCl_3_) δ 159.89 (C=O), 155.54 (C_4_), 130.50, 130.04 (C_2_, C_6_), 128.96, 128.86 (C_1_), 114.25 (C_5_), 114.09 (C_3_), 75.37 (OCH), 55.65 (OCH_3_), 22.35 (CH_3_).

The compound *O*-(3-Methoxybenzyl)hydroxylamine (**17**) was synthesized from the compound 3 (1 equiv.) according to the general procedure. Off-yellow oil (96.4 mg, 89%). R*_f_* = 0.11 (CH_2_Cl_2_). ^1^H NMR (400 MHz, CDCl_3_) δ 7.31–7.27 (m, 1H, Ar), 6.97–6.83 (m, 3H, Ar), 5.41 (s, 2H, NH_2_), 4.68 (s, 2H, OCH_2_), 3.82 (s, 3H, CH_3_). ^13^C NMR (200 MHz, CDCl_3_) δ 161.33 (C_3_), 140.27 (C_1_), 130.50 (C_5_), 121.52 (C_6_), 114.67, 114.59 (C_2_, C_4_), 78.83 (OCH_2_), 55.67 (OCH_3_) [43].

The compound Methyl 4-((aminooxy)methyl)-3,5-dichlorobenzoate (**18**) was synthesized from the compound 4 (200.0 mg, 0.53 mmol) according to the general procedure (18 h), to afford an off-yellow oil (46.8 mg, 36%), which was used in the next step without further purification.

The compound 4-((Aminooxy)methyl)phenol (**19**) was synthesized from the compound **5** (1 equiv.) according to the general procedure. Off-yellow oil (99.2 mg, 99%). R*_f_* = 0.14 (CH_2_Cl_2_). ^1^H NMR (600 MHz, DMSO) δ 7.11 (d, *J* = 8.4 Hz, 2H, Ar), 6.73 (d, *J* = 8.5 Hz, 2H, Ar), 4.75 (broad s, 3H, NH_2,_ OH), 4.43 (s, 2H, OCH_2_). ^13^C NMR (100 MHz, CD_3_OD) δ 160.14 (C_1_), 132.50 (C_3_, C_5_), 124.86 (C_4_), 116.63 (C_2_, C_6_), 78.11 (OCH_2_) [44].

The compound ***O*-(Pent-4-yn-1-yl)hydroxylamine (20)** was synthesized from the compound **6** (1 equiv., 0.65 mmol) with the addition of 64% *w*/*w* hydrazine monohydrate (1.1 equiv., 0.72 mmol) at 0 °C. The reaction mixture was stirred for 15 min under argon. Then, the reaction solvent Et_2_O (1.6 mL) was added and the reaction was stirred for another 15 min. The formed white precipitate was filtered under ice and washed with Et_2_O. The filtrate was evaporated without vacuum to afford a volatile colorless oil (40 mg, 62%). R*_f_* = 0.5 (AcOEt). ^1^H NMR (400 MHz, CDCl_3_) δ 5.37 (s, 2H, ΝH_2_), 3.75 (t, *J* = 6.2 Hz, 2H, OCH_2_), 2.27 (td, *J* = 7.1, 2.7 Hz, 2H, C*H_2_*C≡CH), 1.96 (t, *J* = 2.7 Hz, 1H, C≡CH), 1.81 (ddd, *J* = 7.1, 6.1, 0.9 Hz, 2H, CH_2_CH_2_). ^13^C NMR (100 MHz, CDCl_3_) δ 84.0 (C_4_), 74.4 (C_1_), 68.7 (C_5_), 27.4 (C_2_), 15.3 (C_3_) [45].

The compound *O*-(But-3-yn-1-yl)hydroxylamine (**21**) was synthesized from the compound 7 (1 equiv.) according to the procedure used for the compound 20 to afford a volatile off-yellow oil (62.3 mg, 79%). R*_f_* = 0.6 (AcOEt). ^1^H NMR (400 MHz, CDCl_3_) δ 5.46 (s, 2H, NH_2_), 3.78 (t, *J* = 6.6 Hz, 2H, OCH_2_), 2.51 (td, *J* = 6.6, 2.7 Hz, 2H, CH_2_C≡CH), 1.99 (t, *J* = 2.7 Hz, 1H, C≡CH). ^13^C NMR (125 MHz, D_2_O) δ 80.4 (C_3_), 72.9 (C_1_), 70.9 (C_4_), 17.6 (C_2_) [45].

The compound *O*-(Prop-2-yn-1-yl)hydroxylamine (**22**) was synthesized from the compound 8 (1 equiv.) according to the procedure used for the compound 20 to afford a volatile colorless oil (26.6 mg, 40%). R*_f_* = 0.3 (AcOEt). ^1^H NMR (400 MHz, CDCl_3_) δ 5.59 (s, 2H, NH_2_), 4.30 (d, *J* = 2.3 Hz, 2H, CH_2_), 2.46 (s, 1H, C≡CH). ^13^C NMR (125 MHz, CD_3_OD) δ 77.3 (C_2_), 75.2 (C_3_), 39.3 (C_1_) [45].

The compound Methyl 4-((aminooxy)methyl)-2-chlorobenzoate (**23**) was synthesized from the compound 9 (1 equiv., 0.29 mmol) in MeOH (1.0 mL) with the slow addition of 64% *w*/*w* hydrazine monohydrate (2 equiv., 0.90 mmol) and the reaction was stirred under argon in rt for 2 h. The formed white solid was filtered and washed with MeOH and the filtrate was evaporated to afford an off-yellow oil (60 mg, 96.5%). R*_f_* = 0.14 (CH_2_Cl_2_). ^1^H NMR (600 MHz, CDCl_3_) δ 8.04 (d, *J* = 1.6 Hz, 1H, Ar), 7.94 (dd, *J* = 7.9, 1.6 Hz, 1H, Ar), 7.53 (d, *J* = 8.0 Hz, 1H, Ar), 5.58–5.56 (m, 2H, NH_2_), 4.86 (s, 2H, OCH_2_), 3.93 (d, *J* = 0.8 Hz, 3H, CH_3_). ^13^C NMR (75 MHz, CDCl_3_) δ 165.9 (C-O), 142.5 (C_1_), 133.4 (C_4_), 132.3 (C_2_), 130.1 (C_3_), 128.6 (C_6_), 128.2 (C_5_), 73.9 (OCH_2_), 51.5 (OCH_3_).

The compound Methyl 4-((aminooxy)methyl)-2-cyanobenzoate (**24**) was synthesized from the compound **10** (1 equiv., 0.30 mmol) in MeOH (1.0 mL) with the slow addition of 64% *w*/*w* hydrazine monohydrate (1.1 equiv., 0.51 mmol) and the reaction was stirred under argon in rt for 2 h. The formed white solid was filtered and washed with MeOH and the filtrate was evaporated to afford an off-yellow oil (49.4 mg, 80%). R*_f_* = 0.15 (CH_2_Cl_2_). ^1^H NMR (400 MHz, CDCl_3_) δ 8.33 (dd, *J* = 1.8, 0.5 Hz, 1H, Ar), 8.24 (ddd, *J* = 8.1, 1.8, 0.4 Hz, 1H, Ar), 7.68–7.61 (m, 1H, Ar), 5.61 (s, 2H, NH_2_), 4.93 (dd, *J* = 0.7, 0.4 Hz, 2H, OCH_2_), 3.96 (d, *J* = 0.3 Hz, 3H, OCH_3_). ^13^C NMR (75 MHz, CDCl_3_) δ 165.9 (C-O), 148.2 (C_1_), 134.4 (C_5_), 133.3 (C_3_), 129.7 (C_4_), 127.7 (C_6_), 115.8 (C-N), 111.3 (C_2_), 75.7 (OCH_2_), 51.5 (OCH_3_).

The compound Methyl 4-((aminooxy)methyl)-3-fluorobenzoate (**25**) was synthesized from the compound 11 (1 equiv., 0.45 mmol) in MeOH (5.2 mL) with the slow addition of 64% w/w hydrazine monohydrate (1.1 equiv., 0.50 mmol) and the reaction was stirred under argon in rt for 2 h. The formed white solid was filtered and washed with MeOH and the filtrate was evaporated to afford an off-yellow oil (60 mg, 99%). R*_f_* = 0.12 (CH_2_Cl_2_). ^1^H NMR (400 MHz, CDCl_3_) δ 7.88–7.79 (m, 1H, Ar), 7.72 (dd, *J* = 10.3, 1.6 Hz, 1H, Ar), 7.55–7.44 (m, 1H, Ar), 5.52 (s, 2H, NH_2_), 4.81 (d, *J* = 1.0 Hz, 2H, OCH_2_), 3.93 (s, 3H, CH_3_). ^13^C NMR (126 MHz, CDCl_3_) δ 166.04, 166.01 (C=O), 161.73, 159.75 (C_3_), 131.84, 131.78 (C_4_), 130.39 (C_1_), 130.35 (C_5_), 130.16, 130.04 (C_6_), 125.39, 125.37 (C_2_), 116.76, 116.57 (OCH_2_), 52.56 (OCH_3_).

The compound *O*-(1-Phenylethyl)hydroxylamine (**26**) was synthesized from the compound 12 (200.0 mg, 0.75 mmol) according to the general procedure (3 h) using MeOH (3 mL) as solvent. White solid (114.8 mg, quantitative yield). ^1^H NMR (400 MHz, CDCl_3_) δ 7.38–7.23 (m, 5H), 4.65 (q, *J* = 6.5 Hz, 1H), 1.41 (d, *J* = 6.6 Hz, 3H) ppm [46].

The compound Methyl 4-((aminooxy)methyl)benzoate (**27**) was synthesized from the compound **13** (1 equiv.) according to the general procedure, to afford an off-yellow oil (89.1 mg, 61.23%). R*_f_* = 0.14 (CH_2_Cl_2_). ^1^H NMR (400 MHz, CDCl_3_) δ 7.55–7.49 (m, 2H, Ar), 7.31–7.27 (m, 2H, Ar), 5.52–5.38 (m, 2H, NH_2_), 4.67 (s, 2H, OCH_2_), 3.96 (s, 3H, OCH_3_). ^13^C NMR (101 MHz, CDCl_3_) δ 165.9 (C-O), 136.6 (C_1_), 131.5 (C_5_, C_3_), 130.0 (C_6_, C_2_), 121.8 (C_4_), 77.0 (OCH_2_), 51.5 (CH_3_) [47].

The compound *O*-(4-Nitrobenzyl)hydroxylamine (**28**) was synthesized from the compound **14** (200.0 mg, 0.67 mmol) according to the general procedure (3 h). Orange oil (110.0 mg, 98%). ^1^H NMR (400 MHz, CDCl_3_) δ 8.22 (d, *J* = 8.7 Hz, 2H), 7.52 (dt, *J* = 8.8, 0.7 Hz, 2H), 5.53 (s, 2H), 4.78 (s, 2H) ppm [48].

#### 3.2.4. Synthesis of *O*-Substituted Hydroxylamines **29**–**31**

The compound 2-Bromo-1-(3,5-difluorophenyl)ethenone (**i**) was synthesized as follows. A solution of Br_2_ (11.25 g, 3.63 mL, 70 mmol, 1.1 equiv.) in CHCl_3_ (90 mL) was slowly added to a solution of 1-(3,5-difluorophenyl)ethanone (10 g, 64 mmol, 1 equiv.) in CHCl_3_ (90 mL) within 6 h at room temperature. After the end of the addition, the reaction was left stirring for another 2 h. The reaction mixture was then diluted with CH_2_Cl_2_ (300 mL) and washed with NaHCO_3_ (sat.) (1 × 250 mL), Na_2_S_2_O_3_ (1 × 250 mL), water (1 × 250 mL), and brine (1 × 250 mL), and the organic layer was dried over anh. Na_2_SO_4_, filtered, and concentrated to dryness to afford 14.5 g of a pale yellow oil. ^1^H NMR analysis showed the presence of a small quantity of the starting material (same R_f_ as the desired product in hexane/EtOAc 9:1) in a 10.7:1 molar ratio (94% purity by weight, 92% yield in bromide) and was used for the next step without further purification. ^1^H NMR (CDCl_3_, 500 MHz) δ 7.52–7.47 (m, 2H, Ar*H*3, Ar*H*5), 7.07 (tt, ^3^*J*_ArH-F_ = 8.3 Hz, ^4^*J*_ArH-H_ = 2.3 Hz, 1H, Ar*H*1), 4.38 (s, 2H, -C*H*_2_Br). ^13^C NMR (CDCl_3_, 63 MHz) δ 189.12 (Ar*C*=O), 163.28 (dd, ^1^*J*_C-F_ = 25.3 Hz, ^3^*J*_C-F_ = 11.86 Hz, Ar*C*2, Ar*C*6), 136.8 (t, ^3^*J*_C-F_ = 7.89 Hz, Ar*C*4), 112.2 (dd, ^2^*J*_C-F_ = 26.2 Hz, ^4^*J*_C-F_ = 9.4 Hz, Ar*C*3, Ar*C*5), 109.57 (t, ^2^*J*_C-F_ = 25.31 Hz, Ar*C*1), 30.24 (*C*H_2_Br). Elemental analysis was not performed. ^1^HNMR data match the ^1^H NMR spectrum reported in a patent.

The compound 2-Amino-1-(3,5-difluorophenyl)ethanone hydrochloride (**ii**) was synthesized as follows. To a solution of crude 2-bromo-1-(3,5-difluorophenyl)ethanone (13.78 g in bromide, 58.6 mmol, 1 equiv.) in CHCl_3_ (70 mL), urotropine was added (9.04 g, 64.5 mmol, 1.1 equiv.). After a short time, heavy precipitation occurred, forming a white slurry which was stirred for 4 h. The precipitate was then filtered off, washed with cold CHCl_3_, and dried under vacuum to afford 20.28 g of the intermediate salt. The product was suspended in absolute ethanol (100 mL) followed by the dropwise addition of aq. HCl (37%) (32 mL) within 15 min. After the addition was complete, a clear solution formed soon after, followed by a white precipitation after 1 h approximately. The resulting suspension was stirred overnight and then the precipitate was filtered off and washed thoroughly with ethanol. The filtrate was evaporated to dryness and the resulting solid was recrystallized by ethanol to afford the title compound as a white solid (8.4 g, 69%). ^1^H NMR (500 MHz, D_2_O) δ 7.62–7.60 (m, 2H, Ar*H*3, Ar*H*5), 7.33 (tt, 1H, ^3^*J*_ArH-F_ = 8.8 Hz ^4^*J_Ar_*_H-H_ = 2.4 Hz, Ar*H*1), 4.67 (s, 2H, C*H*_2_NH_2_). ^13^C NMR (63 MHz, D_2_O) δ 191.69 (Ar*C*=O), 162.77 (dd, ^1^*J*_C-F_ = 249 Hz, ^3^*J*_C-F_ = 12.3 Hz, Ar*C*2,*C*6), 135.72 (t, ^3^*J*_C-F_ = 8.5 Hz, Ar*C4*), 111.36 (dd, ^2^*J*_C-F_ = 17.03 Hz, ^4^*J*_C-F_ = 9.61 Hz, Ar*C*3,*C*5), 110.15 (t, ^2^*J*_C-F_ = 25.8 Hz, Ar*C*1), 45.42 (CH_2_NH_2_). Elemental analysis calcd (%) for C_8_H_8_ClF_2_NO: C, 46.28; H, 3.88; N, 6.75; found: C, 46.01; H, 3.93; N, 6.81.

The compound *tert*-Butyl (2-(3,5-difluorophenyl)-2-oxoethyl)carbamate (**iii**) was synthesized as follows. Boc anhydride (6.92 g, 7.3 mL, 31.7 mmol, 1.5 equiv.) was added portion-wise to a solution of the compound **ii** (4.39 g, 21.1 mmol, 1 equiv.) in a mixture of ΜeOH/H_2_O (1:1, 182 mL), followed by the immediate addition of solid NaHCO_3_ (4.44 g, 53 mmol, 2.5 equiv.). The reaction was stirred for 90 min, at which time TLC confirmed the full consumption of the starting material. The reaction was then poured into 800 mL of cold water and extracted with DCM (4 × 200 mL). The combined organic layers were washed with brine (1 × 300 mL), dried over anh. Na_2_SO_4_, filtered, and evaporated to dryness to afford 5.84 g (quant.) of the title compound which was sufficiently pure by NMR analysis. ^1^H NMR (250 MHz, CDCl_3_) δ 7.48–7.41 (m, 2H, Ar*H*3, Ar*H*5), 7.06 (tt, 1H, _3_*J*_ArH-F_ = 8.35 Hz, ^4^*J*_ArH-H_ = 2.3 Hz, Ar*H*1), 5.43 (br, 1H, N*H*Boc), 4.6 (d, *J* = 4.66 Hz, 2H, C*H*_2_NHBoc), 1.46 (s, 9H, C(C*H*_3_)_3_ NH*Boc*). ^13^C NMR (63 MHz, CDCl_3_) δ 163.3 (dd, ^1^*J*_C-F_ = 252.16 Hz, ^3^*J*_C-F_ = 11.7 Hz, Ar*C*2,*C*6), 155.79 (Ar*C*=O), 146.86 (NH*C*(=O)OtBu), 137.43 (t, ^3^*J*_C-F_ = 7.72 Hz, Ar*C*4), 110.02 (dd, ^2^*J*_C-F_ = 25.75 Hz, ^4^*J*_C-F_ = 8.97 Hz, Ar*C*3,*C*5), 109.34 (t, ^2^*J*_C-F_ = 25.53 Hz, Ar*C*1), 80.24 (-O*C*(CH_3_)_3_), 47.84 (s, *C*H2NHBoc), 28.41 (-OC(*C*H_3_)_3_). Elemental analysis calcd (%) for C_13_H_15_F_2_NO_3_: C, 57.56; H, 5.57; N, 5.16; found: C, 57.74; H, 5.76; N, 5.01.

The compound *tert*-Butyl (2-(3,5-difluorophenyl)-2-hydroxyethyl)carbamate (**iv**) was synthesized as follows. NaBH_4_ (0.96 g, 25 mmol, 1.2 equiv.) was added in 2 portions to an ice-cold solution of **iii** (5.74 g, 21.1 mmol, 1 equiv.) in absolute EtOH (34 mL). The reaction was stirred at 0 °C for 1 h and it was then quenched with water (8 mL) and stirred for an additional 30 min at room temperature. After removal of the volatiles under reduced pressure, the residue was dissolved in DCM (200 mL) and washed with water (2 × 75 mL) and brine (1 × 75 mL) to afford 4.52 g of the desired product as a pale yellow oil which was sufficiently pure by NMR analysis and used in the next step without further purification (Yield: 78%). ^1^H NMR (500MHz, CDCl_3_) δ 6.93–5.89 (m, 2H, Ar*H*3, Ar*H*5), 6.71 (tt, ^3^*J*_ArH-F_ = 8.84 Hz, ^4^*J_Ar_*_H-H_ = 2.06 Hz, Ar*H*1), 4.91 (br, 1H, -N*H*Boc), 4.87–4.77 (m, 1H, ArC*H*CH_2_NHBoc), 3.54–3.12 (m, 1H, ArCHC*H*_2_NHBoc), 3.27–3.17 (m, 1H, ArCHC*H*_2_NHBoc), 1.44 (s, 9H, -C(C*H*_3_)_3_ NH*Boc*). ^13^C NMR (63MHz, CDCl_3_) δ 163.23 (dd, ^1^*J*_C-F_ = 248.7 Hz, ^3^*J*_C-F_ = 12.6 Hz, Ar*C*2, Ar*C*6), 157.59 (NH*C*(=O)O^t^Bu), 146.23 (t, ^3^*J*_C-F_ = 8.43 Hz, Ar*C*4), 108.91 (dd, ^2^*J*_C-F_ = 25.29 Hz, ^4^*J*_C-F_ = 8.67 Hz, Ar*C*3,*C*5), 103.1 (t, ^2^*J*_C-F_ = 25.4 Hz, Ar*C*1), 80.57 (-OC(CH_3_)_3_), 73.58 (Ar*C*HCH_2_NHBoc), 48.51 (ArCH*C*H_2_NHBoc), 28.45 (-OC(CH_3_)_3_). Elemental analysis calcd (%) for C_13_H_17_F_2_NO_3_: C, 57.14; H, 6.27; N, 5.13; found: C, 57.03; H, 6.35; N, 5.08.

The compound *tert*-Butyl (2-(3,5-difluorophenyl)-2-((1,3-dioxoisoindolin-2-yl)oxy)ethyl)carbamate (**v**) was synthesized as follows. A solution of DEAD (3.74 g, 21.5 mmol, 1.3 equiv.) in dry THF (36.7 mL) was added dropwise within 90 min to a mixture of **iv** (4.52 g, 16.5 mmol, 1 equiv.), triphenylphosphine (5.64 g, 21.5 mmol, 1.3 equiv.), and *Ν*-hydroxyphthalimide (3.50 g, 21.5 mmol, 1.3 equiv.) in dry THF (74 mL) at −10 °C. During the addition of DEAD, the color changed abruptly from pale yellow to deep red. The reaction mixture was stirred and allowed to reach room temperature slowly overnight. During this time, the color of the reaction changed to light yellow and TLC confirmed the complete consumption of the starting material. The reaction mixture was evaporated under reduced pressure and the crude mixture was purified by flash column chromatography using hexane/EtOAc 8:2, to provide the desired compound as a colorless oil (6.92 g, 94%). ^1^H NMR (500 MHz, CDCl_3_) δ 7.85–7.71 (m, 4H, *Phth*), 7.12–7.02 (m, 2H, Ar*H*3, Ar*H*5), 6.78 (tt, ^3^*J*_ArH-F_ = 8.9 Hz, ^4^*J*_ArH-H_ = 2.06 Hz, 1H, Ar*H1*), 5.52 (br, 1H, N*H*Boc), 5.32–5.25 (m, 1H, PhC*H*CH_2_NHBoc), 3.65–3.58 (m, 2H, PhCHC*H_2_*NHBoc), 1.42 (s, 9H, -C(C*H_3_*)_3_ NH*Boc*). ^13^C NMR (125 MHz, CDCl_3_) δ 163.89 (*C*=O Phth), 163.05 (dd, ^1^*J*_C-F_ = 249.63 Hz, ^3^*J*_C-F_ =12.56 Hz, Ar*C*2, Ar*C*6), 156.01 (NH*C*(=O)OtBu), 139.93 (t, ^3^*J*_C-F_ = 7.86 Hz, Ar*C*4), 134.94 (*Phth*), 128.91 (*Phth*), 123.95 (*Phth*), 110.67 (dd, ^2^*J*_C-F_ = 18.62 Hz, ^4^*J*_C-F_ = 5.88 Hz, Ar*C*3, Ar*C*5), 104.57 (t, ^2^*J*_C-F_ = 25.19 Hz, Ar*C*1), 87.36 (Ar*C*HCH_2_NHBoc), 79.95 (-O*C*(CH_3_)_3_), 44.31 (ArCH*C*H_2_NHBoc), 28.47 (-OC(*C*H_3_)_3_). Elemental analysis calcd (%) for C_21_H_20_F_2_N_2_O_5_: C, 60.28; H, 4.82; N, 6.70; found: C, 60.41; H, 4.89; N, 6.62.

The compound 2-(2-Amino-1-(3,5-difluorophenyl)ethoxy)isoindoline-1,3-dione (**vi**) was synthesized as follows. **v** (6.39 g, 15.3 mmol, 1 equiv.) was dissolved in 3 M HCl in EtOAc (19.3 mL) and within a few minutes a white precipitate formed. The reaction was stirred for 90 min and then the solid was collected by filtration, washed thoroughly with EtOAc and Et_2_O, and dried under vacuum to afford the title compound (5.08 g, 94%). ^1^H NMR (250 MHz, DMSO-*d*_6_) δ 8.44 (br, 3H, -N*H*_2_) 7.86 (s, 4H, *Phth*), 7.43–7.25 (m, 3H, Ar*H*1, Ar*H*3, Ar*H*5), 5.52 (t, *J* = 6 Hz, 1H, ArC*H*CH_2_NH_2_), 3.67–3.53 (m, 1H, ArCHC*H*_2_NH_2_), 3.44–3.28 (m, 1H, ArCHC*H_2_*NH_2_). ^13^C NMR (63 MHz, DMSO-*d*_6_) δ 163.12 (C=O *Phth*), 161.8 (dd, ^1^*J*_C-F_ = 247.76 Hz, ^3^*J*_C-F_ = 13.22 Hz, Ar*C*2, Ar*C*6), 138.76 (t, ^3^*J*_C-F_ = 7.86 Hz, Ar*C4*), 135.08 (*Phth*), 128.32 (*Phth*), 123.53 (*Phth*), 111.8 (dd, ^2^*J*_C-F_ = 25.81 Hz, ^4^*J*_C-F_ = 8.59 Hz, Ar*C3*, Ar*C5*), 105.01 (t, ^2^*J*_C-F_ = 25.76 Hz, Ar*C*1), 84.20 (Ar*C*HCH_2_NH_2_), 41.22 (ArCH*C*H_2_NH_2_). Elemental analysis calcd (%) for C_16_H_13_ClF_2_N_2_O_3_: C, 54.17; H, 3.69; N, 7.90; found: C, 53.99; H, 3.75; N, 7.81.

The compound *N*-(2-(Aminooxy)-2-(3,5-difluorophenyl)ethyl)furan-2-carboxamide (**29**) was synthesized as follows. To a solution of **vi** (0.35 g, 0.99 mmol, 1 equiv.) and furane-2-carboxylic acid (0.133 g, 1.18 mmol, 1.2 equiv.) in DMF (6.3 mL), TBTU (0.38 g, 1.18 mmol, 1.2 equiv.) was added followed by the dropwise addition of DIPEA (0.41 mL, 2.37 mmol, 2.4 equiv.). After stirring overnight, the reaction was poured into an ice-cold NaHCO_3_ (sat.) solution (70 mL) and the precipitate formed was filtered off, washed with cold water, and dried under over P_2_O_5_. The crude amide was dissolved in a mixture of EtOH/THF (3:1, 10 mL), aq. methylamine 40% (0.39 mL, 4.97 mmol, 5 equiv.) was added, and the reaction was stirred overnight at room temperature. The volatiles were removed under reduced pressure and the residue was dissolved in the minimum amount of ether and was cooled to 0 °C for 1 h. The precipitate formed was filtered off, the filtrate was evaporated to dryness, and the residue was purified by flash column chromatography using hexane/EtOAc/Et_3_N 5:5:0.1 to afford the title compound as a pale yellow oil (0.27 g, 92% over two steps). ^1^H NMR (500 MHz, DMSO-*d*_6_) δ 8.37 (t, *J* = 5.7 Hz, 1H, -N*H*CO), 7.82 (br, 1H, furan*H5*), 7.12 (tt, ^3^*J*_ArH-F_ = 9.2 Hz, ^4^*J*_ArH-F_ = 2 Hz, 1H, Ar*H1*), 7.08 (d, *J* = 3.4, 1H, furan*H*3), 7.03–6.95 (m, 2H, Ar*H*3, Ar*H*5), 6.61 (dd, *J*_1_ = 3.4, *J*_2_ = 1.7 Hz, 1H, furan*H*4), 6.19 (s, 2H, *O*-N*H*_2_), 4.67 (t, *J* = 5.9 Hz, 1H, ArC*H*CH_2_NHCO), 3.48–3.43 (m, 2H, ArCHC*H_2_*NHCO). ^13^C NMR (125 MHz, DMSO-*d*_6_) δ 162.23 (dd, ^1^*J*_C-F_ = 245.8, ^3^*J*_C-F_ = 12.9 Hz, Ar*C*2, Ar*C*6), 157.75 (-NH*C*O), 147.72 (furan*C*2), 145.50 (t, ^3^*J*_C-F_ = 8.3 Hz, Ar*C*4), 145.00 (furan*C*5), 113.40 (furan*C*3), 111.79 (furan*C*4), 109.79 (dd, ^2^*J*_C-F_ = 19.9, ^4^*J*_C-F_ = 5.3 Hz, Ar*C*3, Ar*C*5), 102.71 (t, ^2^*J*_C-F_ = 25.7 Hz, Ar*C*1), 82.95 (Ar*C*HCH_2_NHCO), 42.57 (ArCH*C*H_2_NHCO). Elemental analysis calcd (%) for C_13_H_12_F_2_N_2_O_3_: C, 55.32; H, 4.29; N, 9.93; found: C, 55.44; H, 4.35; N, 10.02.

The compound *N*-(2-(Aminooxy)-2-(3,5-difluorophenyl)ethyl)quinoline-2-carboxamide (**30**) was synthesized as follows. To a solution of **vi** (0.35 g, 0.99 mmol, 1 equiv.) and quinoline-2-carboxylic acid (0.205 g, 1.18 mmol, 1.2 equiv.) in DMF (6.5 mL), TBTU (0.38 g, 1.18 mmol, 1.2 equiv.) was added followed by the dropwise addition of DIPEA (0.41 mL, 2.37 mmol, 2.4 equiv.). After stirring overnight, the reaction was poured into an ice-cold NaHCO_3_ (sat.) solution (50 mL) and the precipitate formed was filtered off, washed with cold water, and dried under over P_2_O_5_. The crude material was dissolved in THF (5 mL), and aq. hydrazine hydrate 55% m/w (0.18 mL, 1.98 mmol, 2 equiv.) was added dropwise. The reaction was stirred for 1 h, at which point a TLC check confirmed the full consumption of the starting material. Water (40 mL) was added and the reaction was extracted with EtOAc (4 × 20 mL), the combined organic layers were washed with brine, dried over anh. Na_2_SO_4_, and evaporated to dryness. The residue was purified by flash column chromatography using hexane/EtOAc/Et_3_N 6:4:0.1 to afford the title compound as a pale yellow oil (0.288 g, 85%). ^1^H NMR (250 MHz, DMSO-*d*_6_) δ 8.88 (t, *J* = 5.68 Hz, 1H, -N*H*CO), 8.57 (d, ^3^*J*_H-H_ = 8.4 Hz, 1H, quin), 8.16–8.11 (m, 2H, quin), 8.09 (d, ^3^*J*_H-H_ = 8.41 Hz, 1H, quin), 7.92–7.86 (m, 1H, quin), 7.76–7.70 (m, 1H, quin), 7.13 (tt, ^3^*J*_H-F_ = 9.25 Hz, ^4^*J*_H-H_ = 2.3 Hz, 1H, Ar*H*1), 7.10–7.05 (m, 2H, Ar*H*3,*H*5), 6.26 (s, 2H, *O*-N*H*_2_), 4.81 (t, *J* = 5.83 Hz, 1H, PhC*H*CH_2_NHCO), 3.67–3.61 (m, 2H, PhCHC*H*_2_NHCO). ^13^C NMR (125 MHz, DMSO-*d*_6_) δ 163.89 (s, -NH*C*O), 162.28 (dd, ^1^*J*_C-F_ = 246.75 Hz, ^3^*J*_C-F_ = 12.92 Hz, Ar*C*2,*C*6), 149.79 (s, quin), 145.92 (s, quin), 145.41 (t, ^3^*J*_C-F_ = 8.54 Hz, Ar*C*4), 137.97 (s, quin), 130.58 (s, quin), 129.12 (s, quin), 128.83 (s, quin), 128.12 (quin, 2C based on HSQC), 128.09 (s, quin), 118.53 (s, quin), 109.81 (dd, ^2^*J*_C-F_ = 19.44 Hz, ^4^*J*_C-F_ = 5.76 Hz, Ar*C3*,*C5*), 102.73 (t, ^2^*J*_C-F_ = 25.97 Hz, Ar*C*1), 82.93 (s, Ar*C*HCH_2_NHCO), 43.14 (s, ArCH*C*H_2_NHCO). Elemental analysis calcd (%) for C_18_H_15_F_2_N_3_O_2_: C, 62.97; H, 4.40; N, 12.24; found: C, 63.11; H, 4.45; N, 12.32.

The compound *N*-(2-(Aminooxy)-2-(3,5-difluorophenyl)ethyl)thiazole-2-carboxamide (**31**) was synthesized as follows. To a solution of **vi** (100 mg, 0.28 mmol, 1 equiv.) and thiazole-2-carboxylic acid (43.6 mg, 0.34 mmol, 1.2 equiv.) in DMF (1.8 mL), TBTU (109 mg, 0.34 mmol, 1.2 equiv.) was added followed by the dropwise addition of DIPEA (0.12 mL, 0.68 mmol, 2.4 equiv.). After stirring overnight, the reaction was poured into an ice-cold NaHCO_3_ (sat.) solution (30 mL) and the precipitate formed was filtered off, washed with cold water, and dried under over P_2_O_5_. The crude material was dissolved in THF (1.4 mL), and aq. hydrazine hydrate 55% m/w (50 μL, 0.56 mmol, 2 equiv.) was added dropwise. The reaction was stirred for 90 min at which point a TLC check confirmed the full consumption of the starting material. Water (30 mL) was added and the reaction was extracted with EtOAc (4 × 15 mL), the combined organic layers were washed with brine, dried over anh. Na_2_SO_4_, and evaporated to dryness. The residue was purified by flash column chromatography using hexane/EtOAc/Et_3_N 5:5:0.1 to afford the title compound as a pale yellow oil (55 mg, 67%). ^1^H NMR (250 MHz, DMSO-*d*_6_) δ 8.76 (t, *J* = 5.6 Hz, 1H, -N*H*CO), 8.04 (d, *J* = 2.8 Hz, 1H, thiaz*H*4), 8.01 (d, *J* = 2.8 Hz, 1H, thiaz*H*5), 7.13 (tt, ^3^*J*_H-F_ = 9.2 Hz, ^4^*J*_H-H_ = 2.1 Hz, 1H, Ar*H*1), 7.06–6.94 (m, 2H, Ar*H*3, Ar*H*5), 6.23 (s, 2H, *O*-N*H*_2_), 4.74 (t, *J* = 5.9 Hz, 1H, ArC*H*CH_2_NH_2_CO-), 3.63–3.46 (m, 2H, ArCHC*H*_2_NHCO). ^13^C NMR (63 MHz, DMSO-*d*_6_) δ 163.54 (s, -NH*C*O), 162.26 (dd, ^1^*J*_C-F_ = 245.7 Hz, ^3^*J*_C-F_ = 13.1 Hz, Ar*C*2, Ar*C*6), 159.12 (thiaz*C*2), 145.28 (t, ^3^*J*_C-F_ = 8.54 Hz, Ar*C*4), 143.89 (thiaz*C*5), 125.82 (thiaz*C*4), 109.85 (dd, ^2^*J*_C-F_ = 15 Hz, ^4^*J*_C-F_ = 8.15 Hz Ar*C*3, Ar*C*5), 102.8 (t, ^2^*J*_C-F_ = 26.7 Hz, Ar*C*1), 59.78 (Ar*C*HCH_2_NHCO), 43.01 (ArCH*C*H_2_NHCO). Elemental analysis calcd (%) for C_12_H_11_F_2_N_3_O_2_S: C, 48.16; H, 3.70; N, 14.04; found: C, 48.32; H, 3.81; N, 14.15.

#### 3.2.5. Synthesis of *N*-Hydroxypyridinedione Oximes **33**–**50**

General procedure:

To a solution of the appropriate hydroxylamine (0.57 mmol, 1.05 equiv.) in abs. EtOH (2 mL), 5-acetyl-1,6-dihydroxy-4-methylpyridin-2(1*H*)-one (B) (0.55 mmol, 1 equiv.) is added and the reaction mixture is stirred at RT, under argon, overnight. Thereafter, the solvent is evaporated under vacuum. The solid residue is triturated with Et_2_O under ice to afford the desired compound as a solid.

The compound 1,6-Dihydroxy-4-methyl-5-(1-(((4(methylthio)benzyl)oxy)imino)ethyl)pyridin-2(1H)-one (**33**) was synthesized from the compound **15** (1.05 equiv.) according to the general procedure. Green solid (100.7 mg, 61%). R*_f_* = 0.25 (AcOEt), m.p. 110–115 °C (dec.). ^1^H NMR (400 MHz, DMSO) δ 7.32–7.23 (m, 4H, Ar), 5.40 (d, *J* = 23.0 Hz, 1H, CHC=ON), 4.96 (d, *J* = 49.1 Hz, 2H, OCH_2_), 2.46 (d, *J* = 3.0 Hz, 3H, SCH_3_), 1.97 (d, *J* = 12.4 Hz, 3H, CH_3_C=N), 1.86 (d, *J* = 13.9 Hz, 3H, CH_3_). ^13^C NMR (101 MHz, DMSO) δ 128.53 (C_2′_, C_6′_), 125.84 (C_3′_, C_5′_), 91.72 (C_3_), 74.37 (OCH_2_), 19.93 (7-CH_3_), 14.80 (4-CH_3_), 14.70 (7-CH_3_). Elemental analysis calcd (%) for C_16_H_18_N_2_O_4_S: C, 57.47; H, 5.43; N, 8.38. Found: C, 57.51; H, 5.47; N, 8.42.

The compound Methyl-4-(1-(((1-(1,2-dihydroxy-4-methyl-6-oxo-1,6-dihydropyridin-3-yl)ethylidene)amino)oxy)ethyl)benzoate (**34**) was synthesized from the compound **16** (1.05 equiv.) according to the general procedure. Green solid (92.7 mg, 80%). R*_f_* = 0.10 (AcOEt), m.p. 130 °C (dec.). ^1^H NMR (600 MHz, DMSO) δ 7.94–7.91 (m, 1H, Ar), 7.78 (d, *J* = 8.3 Hz, 1H, Ar), 7.47 (d, *J* = 8.1 Hz, 1H, Ar), 7.39 (d, *J* = 8.1 Hz, 1H, Ar), 5.60 (d, *J* = 68.7 Hz, 1H, CHC=ON), 5.24 (q, *J* = 17.9, 12.4 Hz, 2H, OCH_2_), 3.84 (d, *J* = 3.9 Hz, 3H, OCH_3_), 2.04 (d, *J* = 6.5 Hz, 3H, CH_3_C=N), 1.75–1.64 (m, 3H, CH_3_). ^13^C NMR (151 MHz, DMSO) δ 166.05 (C_1′_), 154.69 (C_2_, C_6_), 129.07 (C_4′_), 126.84 (C_2′_, C_6′_), 126.25, 125.83 (C_3′_, C_5′_), 91.98 (C_5_), 78.90 (OCHPh), 51.99 (OCH_3_), 21.99 (7-CH_3_), 16.07 (4-CH_3_). Elemental analysis calcd (%) for C_18_H_20_N_2_O_6_: C, 59.99; H, 5.59; N, 7.77. Found: C, 60.03; H, 5.63; N, 7.81.

The compound 1,6-Dihydroxy-5-(1-(((3-methoxybenzyl)oxy)imino)ethyl)-4-methylpyridin-2(1H)-one (**35**) was synthesized from the compound **17** (1.05 equiv.) according to the general procedure. Green solid (96.9 mg, 54%). R*_f_* = 0.20 (AcOEt), m.p. 120–122 °C (dec.). ^1^H NMR (600 MHz, DMSO) δ 7.29–7.19 (m, 2H, Ar), 6.94–6.80 (m, 2H, Ar), 5.42 (d, *J* = 40.4 Hz, 1H, CHC=ON), 5.06 (s, 1H, OCH_2_), 4.93 (s, 1H, OCH_2_), 3.75 (d, *J* = 1.8 Hz, 3H, OCH_3_), 2.02 (s, 2H, CH_3_C=N), 1.97 (s, 1H, CH_3_C=N), 1.88 (d, *J* = 5.8 Hz, 3H, CH_3_). ^13^C NMR (126 MHz, DMSO) δ 158.92 (C_3′_), 158.85 (C_2_), 156.33 (C_6_), 154.32 (C_7_), 146.23 (C_4_), 140.10, 139.66 (C_1′_), 129.01 (C_2′_), 119.39 (C_6′_), 112.71, 112.66, 112.60 (C_4′_, C_5′_), 91.11 (C_3_), 74.03 (OCH_2_Ph), 54.68 (OCH_3_), 19.38 (7-CH_3_), 15.78 (4-CH_3_). Elemental analysis calcd (%) for C_16_H_18_N_2_O_5_: C, 60.37; H, 5.70; N, 8.80. Found: C, 60.40; H, 5.74; N, 8.84.

The compound Methyl 3,5-dichloro-4-((((1-(1,2-dihydroxy-4-methyl-6-oxo-1,6-dihydropyridin-3-yl)ethylidene)amino)oxy)methyl)benzoate (**36**) was synthesized from the compound **18** (46.8 mg, 0.19 mmol) according to the general procedure. Yellow solid (62.5 mg, 89%). R*_f_* = 0.09 (EtOAc/MeOH 3:1), m.p. 117–119 °C (dec.). ^1^H NMR (400 MHz, DMSO-*d*_6_) δ 8.02–7.92 (m, 2H, Ar), 5.69 (s, 1H, H_3_), 5.23 (s, 2H, -CH_2_-), 2.53 (s, 3H, 9-CH_3_), 1.77 (d, *J* = 2.2 Hz, 3H, 4-CH_3_), 1.76 (d, *J* = 3.4 Hz, 3H, 7-CH_3_) ppm. ^13^C NMR (151 MHz, DMSO-*d*_6_) δ 154.30 (C_8_), 153.90 (C_6_), 151.64 (C_2_) 151.87 (C_7_), 147.33 (C_2′, 6′_), 133.86 (C_4′_), 131.88 (C_3′, 5′_), 130.03 (C_1′_), 128.34 (C_5_), 127.00 (C_3_), 91.87 (C_4_), 79.56 (C_9_) 77.07 (-CH_2_-), 21.33 (4-CH_3_), 18.64 (7-CH_3_) ppm. Elemental analysis calcd (%) for C_17_H_16_Cl_2_N_2_O_6_: C, 49.18; H, 3.88; N, 6.75; found: C, 49.16; H, 3.89; N, 6.77.

*N*-(2-(3,5-Difluorophenyl)-2-(((1-(1,2-dihydroxy-4-methyl-6-oxo-1,6-dihydropyridin-3-yl)ethylidene)amino)oxy)ethyl)furan-2-carboxamide (37) was synthesized from the compound **29** (1.05 equiv.) according to the general procedure. Blue solid (19 mg, 44%). R*_f_* = 0.25 (AcOEt), m.p. 118–120 °C (dec.). ^1^H NMR (600 MHz, DMSO) δ 8.43 (t, *J* = 6.1 Hz, 1H, C^5^H-O), 7.81 (d, *J* = 1.7 Hz, 1H, C^3^*H*-CH-CHO), 7.17–7.08 (m, 2H, Ph), 7.04–7.00 (m, 1H, Ph), 6.60 (dd, *J* = 3.5, 1.8 Hz, 1H, C^4^*H*-CHO), 5.58–5.41 (m, 1H, CHC=ON), 5.28 (t, *J* = 6.5 Hz, 1H, CH_2_C^2^*H*O), 3.59 (dd, *J* = 13.4, 5.3 Hz, 2H, C^1^*H_2_*CHO), 2.27 (s, 2H, C^2^H_3_), 2.08 (d, *J* = 2.6 Hz, 3H, C^4^H_3_), 1.76 (s, 1H, C^2^H_3_). ^13^C NMR (101 MHz, DMSO) δ 158.49, 114.04, 148.00, 148.00, 110.64, 162.29, 148.00, 110.64, 114.04, 158.49, 158.49, 137.22. Elemental analysis calcd (%) for C_21_H_19_F_2_N_3_O_6_: C, 56.38; H, 4.28; N, 9.39. Found: C, 56.42; H, 4.32; N, 9.43.

The compound 5-(1-(((3,5-Difluorophenyl)(pyridin-2-yl)methoxy)imino)ethyl)-1,6-dihydroxy-4-methylpyridin-2(1H)-one (**38**) was synthesized from the compound **32** (1.05 equiv.) according to the general procedure. Green solid (46 mg, 44%). R*_f_* = 0.25 (AcOEt), m.p. 102–104 °C (dec.). ^1^H NMR (600 MHz, DMSO) δ 8.53 (dt, *J* = 4.7, 1.6 Hz, 1H, C^6^H pyridine), 7.84 (td, *J* = 7.7, 1.9 Hz, 1H, C^4^H pyridine), 7.58 (dd, *J* = 7.9, 1.2 Hz, 1H, C^5^H pyridine), 7.31 (dddt, *J* = 8.4, 5.9, 4.6, 2.0 Hz, 1H, C^3^H pyridine), 7.18–7.12 (m, 3H, Ph), 6.24 (s, 1H,PyCHO), 5.44 (s, 1H, CHC=ON), 2.29 (s, 2H, CH_3_C=N), 2.09 (s, 1H, CH_3_C=N), 1.96 (dd, *J* = 11.3, 2.2 Hz, 1H, CH_3_), 1.75 (s, 2H, CH_3_). ^13^C NMR (151 MHz, DMSO) δ 149.53 (C_4_, C_6-pyr_), 137.74 (C_4-pyr_, C_1-Ph_), 123.53 (C_3-oyr_, C_5-pyr_), 121.78 (C_3-Ph_, C_5-Ph_), 110.67 (C_4-Ph_), 110.39 (C_6-Ph,_ C_2-Ph_), 103.45 (C_5_), 91.51 (C_3_), 85.54, 85.13 (NO*CH*), 24.18 (7-CH_3_),19.91 (4-CH_3_), 16.70 (7-CH_3_). Elemental analysis calcd (%) for C_20_H_17_F_2_N_3_O_4_: C, 59.85; H, 4.27; N, 10.47. Found: C, 59.89; H, 4.30; N, 10.50.

The compound 1,6-Dihydroxy-5-(1-(((4-hydroxybenzyl)oxy)imino)ethyl)-4-methylpyridin-2(1H)-one (**39**) was synthesized from the compound **19** (1.05 equiv.) according to the general procedure. Hydroscopic red solid (119.9 mg, 97%). R*_f_* = 0.25 (AcOEt), m.p. 98–100 °C (dec.). ^1^H NMR (600 MHz, DMSO) δ 7.17–7.08 (m, 2H, Ar), 6.73 (q, *J* = 7.9, 7.3 Hz, 2H, Ar), 6.10–5.16 (m, 1H, CHC=ON), 5.03–4.76 (m, 2H, OCH_2_), 2.06–1.67 (m, 6H, CH_3_C=N, CH_3_). ^13^C NMR (101 MHz, DMSO) δ 156.00 (C_2_), 129.43 (C_4′_), 127.82 (C_1′_), 114.80(C_2′_, C_6′_), 114.72, 114.58 (C_3′_, C_5′_), 64.72 (C_3_), 62.56 (OCH_2_), 14.97 (4-CH_3_, 7-CH_3_). Elemental analysis calcd (%) for C_15_H_16_N_2_O_5_: C, 59.21; H, 5.30; N, 9.21. Found: C, 59.25; H, 5.34; N, 9.25.

The compound 1,6-Dihydroxy-4-methyl-5-(1-((pent-4-yn-1-yloxy)imino)ethyl)pyridin-2(1H)-one (**40**) was synthesized from the compound **20** (1.05 equiv.) according to the general procedure. Green solid (21.2 mg, 28%). R*_f_* = 0.30 (1:1 AcOEt:MeOH), mp: 93–95 °C (dec.). ^1^H NMR (600 MHz, DMSO) δ 5.65–5.45 (m, 1H, CHC=ON), 4.13–3.92 (m, 2H, OCH_2_), 2.33 (d, *J* = 11.4 Hz, 2H, CH_3_C=N), 2.26 (s, 1H, CH_3_C=N), 2.21 (ddd, *J* = 35.2, 7.2, 2.7 Hz, 2H, CH_2_C*H_2_*CH_2_), 2.01 (d, *J* = 17.1 Hz, 2H, CH_2_C≡C), 1.98–1.87 (m, 3H, CH_3_), 1.80–1.77 (m, 1H, C≡C). ^13^C NMR (151 MHz, DMSO) δ 71.69 (C_3_), 59.74 (C_1′_), 40.26 (C_5′_), 28.70 (C_2′_), 25.90 (C_3′_), 20.47 (7-CH_3_), 16.44 (4-CH_3_), 14.93 (7-CH_3_). Elemental analysis calcd (%) for C_13_H_16_N_2_O_4_: C, 59.08; H, 6.10; N, 10.60. Found: C, 59.12; H, 6.14; N, 10.64.

The compound 5-(1-((But-3-yn-1-yloxy)imino)ethyl)-1,6-dihydroxy-4-methylpyridin-2(1H)-one (**41**) was synthesized from the compound **21** (1.05 equiv.) according to the general procedure. Green solid (27 mg, 24%). R*_f_* = 0.30 (1:1 AcOEt:MeOH), m.p. 86–89 °C (dec.). ^1^H NMR (600 MHz, DMSO) δ 5.58–5.42 (m, 1H, CHC=ON), 4.08 (s, 1H, OC*H*H), 3.96 (d, *J* = 6.7 Hz, 1H, OCH*H*), 2.53–2.45 (m, 2H, C*H_2_*C≡CH), 2.33 (s, 1H, C≡CH), 2.01 (s, 2H, CH_3_C=N), 1.98 (s, 3H, CH_3_), 1.91 (s, 1H, CH_3_C=N). ^13^C NMR (151 MHz, DMSO) δ 71.73 (C_3_), 70.31 (C_1′_), 24.81 (C_4′_), 24.36 (C_3′_), 19.56, 19.42 (C_2′_), 18.75 (7-CH_3_), 18.36 (4-C_3_), 15.57 (7-CH_3_). Elemental analysis calcd (%) for C_12_H_14_N_2_O_4_: C, 57.59; H, 5.64; N, 11.19. Found: C, 57.63; H, 5.68; N, 11.23.

The compound 1,6-Dihydroxy-4-methyl-5-(1-((prop-2-yn-1-yloxy)imino)ethyl)pyridin-2(1H)-one (**42**) was synthesized from the compound **22** (1.05 equiv.) according to the general procedure. Green solid (32.3 mg, 54%). R*_f_* = 0.30 (1:1 AcOEt:MeOH), m.p. 78–80 °C (dec.). ^1^H NMR (600 MHz, DMSO) δ 5.65–5.50 (m, 1H, CHC=ON), 3.45 (m, 2H, CH_2_), 2.48 (s, 1H, C≡CH), 2.36 (s, 3H, CH_3_C=N), 2.28 (s, 3H, CH_3_). ^13^C NMR (151 MHz, DMSO) δ 106.25 (C_3_), 56.77 (C_1′_), 24.41 (C_3′_), 19.02 (7-CH_3_), 11.75 (4-CH_3_). Elemental analysis calcd (%) for C_11_H_12_N_2_O_4_: C, 55.93; H, 5.12; N, 11.86. Found: C, 55.97; H, 5.16; N, 11.90.

The compound 2′-Chloro-4′-((((1′-(1,2-dihydroxy-4-methyl-6-oxo-1,6-dihydropyridin-5-yl)ethylidene)amino)oxy)methyl)methyl benzoate (**43**) was synthesized from the compound **23** (1.05 equiv.) according to the general procedure. Green solid (86.4mg, 50.8%). R*_f_* = 0.10 (AcOEt), m.p. 155 °C (dec.). ^1^H NMR (400 MHz, DMSO) δ 7.99–7.73 (m, 2H, Ar), 7.75–7.51 (m, 1H, Ar), 5.53 (s, 1H, H_3_), 5.28–5.07 (s, 2H, OCH_2_), 3.86 (s, *J* = 3.4 Hz, 3H, OCH_3_), 2.30 (s, *J* = 14.3, 3.7 Hz, 3H, CH_3_), 2.14–1.98 (s, 3H, CH_3_). ^13^C NMR (101 MHz, DMSO) δ 165.60 (C- O), 157.96 (C_2_), 156.6 (C_6_), 148.82 (C_7_), 146.8 (C_4_), 142.63 (C_1′_), 133.55 (C_4′_), 130.88 (C_2′_), 130.10 (C_3′_), 128.31 (C_6′_), 120.78 (C_5′_), 108.78 (C_5_), 98.78 (C_3_), 71.79 (OCH_2_Ph), 53.09 (OCH_3_), 20.67 (7- CH_3_), 17.59 (4- CH_3_). Elemental analysis calcd (%) for C_17_H_17_ClN_2_O_6_: C, 53.62; H, 4.50; N, 7.36. Found: C, 53.66; H, 4.53; N, 7.38.

The compound 2′-Cyano-4′-((((1′-(1,2-dihydroxy-4-methyl-6-oxo-1,6-dihydropyridin-5-yl)ethylidene)amino)oxy)methyl)methyl benzoate (**44**) was synthesized from the compound **24** (1.05 equiv.) according to the general procedure. Blue solid (45mg, 55.6%). R*_f_* = 0.10 (AcOEt), m.p. 115 °C. ^1^H NMR (500 MHz, DMSO) δ 8.37–8.16 (m, 2H, Ar), 7.89–7.62 (m, 1H, Ar), 5.47 (s, *J* = 59.6 Hz, 1H, H_3_), 5.35–5.14 (s, 2H, OCH_2_), 3.88 (s, *J* = 3.0 Hz, 3H, OCH_3_), 2.29 (s, 1H, CH_3_), 2.07 (s, *J* = 8.4 Hz, 1H, CH_3_), 2.01–1.84 (s, 2H, CH_3_), 1.82–1.65 (s, 2H, CH_3_). ^13^C NMR (101 MHz, DMSO) δ 165.60 (C- O), 157.96 (C_2_), 156.6 (C_6_), 148.82 (C_7_), 146.8 (C_4_), 142.63 (C_1′_), 133.55 (C_4′_), 130.88 (C_2′_), 130.10 (C_3′_), 128.31 (C_6′_), 120.78 (C_5′_), 117.88 (C-N), 108.78 (C_5_), 98.78 (C_3_), 71.79 (OCH_2_), 53.09 (OCH_3_), 20.67 (7-CH_3_), 17.59 (4-CH_3_). Elemental analysis calcd (%) for C_18_H_17_N_3_O_6_: C, 58.22; H, 4.61; N, 11.32. Found: C, 58.24; H, 4.64; N, 11.36.

The compound Methyl 4-((((1-(1,2-dihydroxy-4-methyl-6-oxo-1,6-dihydropyridin-3-yl)ethylidene)amino)oxy)methyl)-3-fluorobenzoate (**45**) was synthesized from the compound **25** (1.05 equiv.) according to the general procedure. Green solid (131.3 mg, 46%). R*_f_* = 0.05 (AcOEt), m.p. 130 °C (dec.). ^1^H NMR (600 MHz, DMSO) δ 7.85 (t, *J* = 7.5 Hz, 1H, Ar), 7.74–7.68 (m, 1H, Ar), 7.65–7.52 (m, 1H, Ar), 5.71–5.30 (m, 1H, CHC=ON), 5.33–5.14 (m, 2H, OCH_2_), 2.34–2.27 (m, 1H, CH_3_C=N), 2.10–2.03 (m, 2H, CH_3_C=N), 2.02–1.88 (m, 3H, CH_3_). ^13^C NMR (151 MHz, DMSO) δ 133.68 (C_1′_), 133.45 (C_3′_), 130.17 (C_4′_), 129.93 (C_2′_), 129.23 (C_6′_), 128.06 (C_5′_), 110.23 (C_3_), 109.06 (C_5_), 73.66, 73.19, 72.95, 72.72 (OCH_2_), 25.59 (OCH_3_), 16.91 (7-CH_3_), 15.74 (4-CH_3_). Elemental analysis calcd (%) for C_17_H_17_FN_2_O_6_: C, 56.04; H, 4.70; N, 7.69. Found: C, 56.08; H, 4.74; N, 7.72.

The compound 1,6-Dihydroxy-4-methyl-5-(1-((1-phenylethoxy)imino)ethyl)pyridin-2(1*H*)-one (**46**) was synthesized from the compound **26** (100.0 mg, 0.73 mmol) according to the general procedure. Beige solid (135.0 mg, 67%). R*_f_* = 0.08 (EtOAc/MeOH 3:1), m.p. 130–132 °C. ^1^H NMR (500 MHz, DMSO-*d*_6_) δ 8.13–8.03 (m, 5H, Ar), 5.29–5.02 (m, 1H, H_9_), 2.88 (s, 3H, H_8_), 2.72 (s, 3H, 4-CH_3_), 1.74 (d, *J* = 2.4 Hz, 3H, H_10_) ppm. ^13^C NMR (101 MHz, DMSO-*d*_6_) δ 140.87 (C_2_), 137.78 (C_7_), 136.62 (C_6_), 125.89 (C_1′_), 123.78 (C_4′_), 123.67 (C_2′,6′_), 122.92 (C_3′,5′_), 120.28 (C_5_), 119.90 (C_3_), 118.23 (C_4_), 99.82 (C_9_), 67.78 (C_8_), 34.67 (C_4_), 30.26 (C_10_) ppm. Elemental analysis calcd (%) for C_16_H_18_N_2_O_4_: C, 63.56; H, 6.00; N, 9.27; found: C, 63.57; H, 6.03; N, 9.28.

The compound 4′-((((1′-(1,2-Dihydroxy-4-methyl-6-oxo-1,6-dihydropyridin-5-yl)ethylidene)amino)oxy)methyl)methyl benzoate (**47**) was synthesized from the compound **27** (1.05 equiv.) according to the general procedure. Green solid (86.4 mg, 50.8%). R*_f_* = 0.10 (AcOEt), m.p. 120 °C (dec.). ^1^H NMR (600 MHz, DMSO) δ 7.97–7.90 (m, 2H, Ar), 7.52–7.39 (m, 2H, Ar), 5.44 (s, *J* = 46.3 Hz, 1H, H_3_), 5.21–5.01 (s, 2H, OCH_2_), 3.85 (s, 3H, OCH_3_), 2.07–1.93 (s, 3H, CH_3_), 1.91–1.77 (s, 3H, CH_3_). ^13^C NMR (151 MHz, DMSO) δ 166.33 (C-O), 156.79 (C_2_), 154.39 (C_6_), 153.8 (C_7_), 146.70 (C_4_), 129.36 (C_1′_), 128.08 (C_3′_, C_5′_), 127.81 (C_2′_,C_6′_), 126.57 (C_4′_), 108.16 (C_5_), 91.67 (C_3_), 74.03 (OCH_2_Ph), 52.31 (OCH_3_), 20.18 (7-CH_3_), 19.83 (4-CH_3_), 16.27 (7-CH_3_). Elemental analysis calcd (%) for C_17_H_18_N_2_O_6_: C, 58.96; H, 5.24; N, 8.09. Found: C, 58.98; H, 5.27; N, 8.10.

The compound 1,6-Dihydroxy-4-methyl-5-(1-(((4-nitrobenzyl)oxy)imino)ethyl)pyridin-2(1H)-one (**48**) was synthesized from the compound **28** (110.0 mg, 0.65 mmol) according to the general procedure. The brownish residual solid was also triturated with *n*-pentane to afford the title compound as a brown solid (212.4 mg, 97%). R*_f_* = 0.05 (EtOAc/MeOH 3:1), m.p. 105–107 °C. ^1^H NMR (400 MHz, DMSO-*d*_6_) δ 8.22 (d, *J* = 8.6 Hz, 2H, Ar), 7.62 (d, *J* = 8.8 Hz, 2H, Ar), 5.47 (s, 1H, H_3_), 5.24 (s, 2H, -CH_2_-), 2.06 (s, 3H, 7-CH_3_), 1.86 (s, 3H, 4-CH_3_) ppm. ^13^C NMR (101 MHz, DMSO-*d*_6_) δ 155.30 (C_2_), 153.79 (C_6_), 153.50 (C_7_), 146.99 (C_4′_), 128.40 (C_3′, 5′_), 127.03 (C_2′, 6′_), 123.56 (C_1′_), 110.39 (C_5_), 90.96 (C_3_), 73.30 (C_4_), 59.76 (-CH_2_-), 23.32 (7-CH_3_), 19.54 (4-CH_3_) ppm. Elemental analysis calcd (%) for C_15_H_15_N_3_O_6_: C, 54.05; H, 4.54; N, 12.61; found: C, 54.10; H, 4.53; N, 12.65.

The compound *N*-(2-(3,5-Difluorophenyl)-2-(((1-(1,2-dihydroxy-4-methyl-6-oxo-1,6-dihydropyridin-3-yl)ethylidene)amino)oxy)ethyl)quinoline-2-carboxamide (**49**) was synthesized from the hydroxylamine 30 (60.0 mg, 0.17 mmol) according to the general procedure. Beige solid (36.4 mg, 41%). R*_f_* = 0.11 (EtOAc/MeOH 3:1), m.p. 108–110 °C (dec.). ^1^H NMR (400 MHz, DMSO-*d*_6_) δ 8.57 (d, *J* = 8.4 Hz, 1H, Ar), 8.19–8.04 (m, 3H, Ar), 7.95–7.82 (m, 1H, Ar), 7.73 (t, *J* = 7.5 Hz, 1H, Ar), 7.14 (dd, *J* = 21.7, 8.8 Hz, 3H, Ar), 5.44 (s, 1H, H3), 5.40 (t, *J* = 7.5 Hz, 1H, -OCH), 4.98 (d, *J* = 7.5 Hz, 2H, -CH_2_NH-), 2.28 (s, 2H, -CH_3_), 2.12 (s, 1H, -CH_3_), 1.79 (s, 1H, 4-CH_3_), 1.23 (s, 2H, 4-CH_3_) ppm. ^13^C NMR (101 MHz, DMSO-*d*_6_) δ 159.42 (C=O), 140.38 (C_2_), 138.89 (C=N), 127.56 (C_6_), 124.56 (C_3′,5′_), 123.29 (C_8′_), 123.21 (C_9′_), 123.19 (C_16′_), 122.40 (C_4′_), 122.31 (C_2′,6′_), 122.28 (C_1′_), 122.10 (C_9′_), 121.99 (C_15′_), 121.68 (C_13′_), 121.45 (C_12′_), 121.41 (C_11′_), 121.30 (C_10′_), 111.89 (C_4_), 110.27 (CO), 109.34 (C_3_), 109.20 (C_5_) 99.35 (CNH), 67.89 (4-CH_3_), 26.50 (-CH_3_) ppm. Elemental analysis calcd (%) for C_26_H_22_F_2_N_4_O_5_: C, 61.42; H, 4.36; N, 11.02; found: C, 61.43; H, 4.37; N, 11.03.

The compound *N*-(2-(3,5-Difluorophenyl)-2-(((1-(1,2-dihydroxy-4-methyl-6-oxo-1,6-dihydropyridin-3-yl)ethylidene)amino)oxy)ethyl)thiazole-2-carboxamide (**50**) was synthesized from the hydroxylamine 31 (132.0 mg, 0.44 mmol) according to the general procedure. Green solid (69.3 mg, 34%). R*_f_* = 0.11 (EtOAc/MeOH 3:1), m.p. 117–119 °C (dec.). ^1^H NMR (400 MHz, DMSO-*d*_6_) δ 8.11–7.95 (m, 3H, Ar), 7.88 (dd, *J* = 6.0, 3.3 Hz, 1H, Ar), 7.19–7.10 (m, 1H, Ar), 5.47–5.41 (m, 1H, -CHO), 5.38–5.32 (m, 2H, -CH_2_NH-), 2.30 (s, 3H, -CH_3_), 2.07 (s, 3H, 4-CH_3_) ppm. ^13^C NMR (101 MHz, DMSO-*d*_6_) δ 149.56 (C_2_), 145.70 (C=O), 141.29 (C=N), 140.16 (C_6_), 129.28 (C_8′_), 128.56 (C_3′,5′_), 123.29 (C_2′,6′_), 123.18 (C_1′_), 122.89 (C_4′_), 121.86 (C_10′_), 121.62 (C_11′_), 120.26 (C_5_), 120.10 (C_4_), 118.90 (C_3_), 91.83 (CO), 79.72 (CNH), 30.16 (4-CH_3_), 23.89 (CH_3_) ppm. Elemental analysis calcd (%) for C_20_H_18_F_2_N_4_O_5_S: C, 51.72; H, 3.91; N, 12.06; found: C, 51.73; H, 3.96; N, 12.09.

#### 3.2.6. Synthesis of 6-Substituted 2,4-Diaminopyrimidines **51**–**54**

General procedure:

To a flask containing the stirring corresponding alcohol (7.4 equiv.), NaH (1.2 equiv.) is added slowly and the mixture is stirred at 150 °C for 1.5 h. Then, 6-chloropyrimidine-2,4-diamine is added (1 equiv.) and the resulting mixture is stirred at 180 °C for an extra 2–16 h. H_2_O (30 mL) is added and extracted from EtOAc (3 × 30 mL). The combined organic phases are washed with brine (3 × 30 mL), dried over anh. Na_2_SO_4_, filtered, and concentrated. The resulting crude mixture is purified by flash column chromatography.

The compound 6-(3-Methoxyphenoxy)pyrimidine-2,4-diamine (**51**) was synthesized from (3-methoxyphenyl)phenol (7.4 equiv.) according to the general procedure (16 h) using CH_2_Cl_2_/EtOAc 50% to 100% as the eluent. Beige solid (710 mg, 89%). R*_f_* = 0.24 (AcOEt). ^1^H NMR (400 MHz, DMSO) δ 7.28 (t, *J* = 8.5 Hz, 1H, Ph), 6.76 (ddd, *J* = 8.4, 2.3, 1.1 Hz, 1H, Ph), 6.67–6.63 (m, 2H, Ph), 6.22 (s, 2H, NH_2_), 5.95 (s, 2H, NH_2_), 5.04 (s, 1H, CHCNH_2_), 3.74 (s, 3H, OCH_3_). ^13^C NMR (101 MHz, DMSO) δ 170.22 (C_6_), 166.48 (C_4_), 163.34 (C_2_), 160.30 (C_1′_), 154.48 (C_3′_), 130.01 (C_2′_), 113.50 (C_4′_), 110.28 (C_5′_), 107.32 (C_6′_), 77.27 (C_5_), 55.28 (OCH_3_). Elemental analysis calcd (%) for C_11_H_12_N_4_O_2_: C, 56.89; H, 5.21; N, 24.12. Found: C, 56.92; H, 5.24; N, 24.15.

The compound 6-(2,4,5-Trichlorophenoxy)pyrimidine-2,4-diamine (**52**) was synthesized from (2,4,5-trichlorophenyl)phenol (7.5 equiv.) according to the general procedure (16 h) using CH_2_Cl_2_/EtOAc 50% to 100% as the eluent. Beige solid (327.4 mg, 98%). R*_f_* = 0.25 (AcOEt). ^1^H NMR (400 MHz, DMSO) δ 7.97 (s, 1H, Ph), 7.68 (s, 1H, Ph), 6.34 (s, 2H, NH_2_), 6.04 (s, 2H, NH_2_), 5.20 (s, 1H, CHCNH_2_). ^13^C NMR (101 MHz, DMSO) δ 168.86 (C_6_), 166.58 (C_4_), 162.98 (C_2_), 148.59 (C_1′_), 130.91, 130.59 (C_5′_), 130.27 (C_3′_), 128.04 (C_4′_), 126.44 (C_2′_), 126.02 (C_6′_), 76.76 (C_5_). Elemental analysis calcd (%) for C_10_H_7_Cl_3_N_4_O: C, 39.31; H, 2.31; N, 18.34. Found: C, 39.35; H, 2.35; N, 18.38.

The compound 6-(2,4-Dichlorophenoxy)pyrimidine-2,4-diamine (**53**) was synthesized from (2,4-dichlorophenyl)phenol (7.5 equiv.) according to the general procedure (16 h) using CH_2_Cl_2_/EtOAc 50% to 100% as the eluent. White solid (455.4 mg, 49%). R*_f_* = 0.33 (AcOEt). ^1^H NMR (500 MHz, DMSO) δ 6.88 (d, *J* = 2.5 Hz, 1H, Ph), 6.61 (dd, *J* = 8.7, 2.6 Hz, 1H, Ph), 6.46 (d, *J* = 8.7 Hz, 1H. Ph), 5.47 (s, 2H, NH_2_), 5.17 (s, 2H, NH_2_), 4.30 (s, 1H, CHCNH_2_). ^13^C NMR (126 MHz, DMSO) δ 169.19 (C_6_), 166.54 (C_4_), 163.08 (C_2_), 148.18 (C_1′_), 129.59, 128.37, 127.49 (C_3′,_ C_4′_, C_5′_), 125.76 (C_6′_), 76.67 (C_5_). Elemental analysis calcd (%) for C_10_H_8_Cl_2_N_4_O: C, 44.30; H, 2.97; N, 20.67. Found: C, 44.34; H, 3.01; N, 20.71.

The compound 6-((4-Methoxybenzyl)oxy)pyrimidine-2,4-diamine (**54**) was synthesized from 4-methoxybenzyl alcohol (1.9 mL, 15.36 mmol) according to the general procedure (3 h) using CH_2_Cl_2_/EtOAc 50% to 100% as the eluent. White solid (435.4 mg, 85%). R*_f_* = 0.32 (EtOAc), m.p. > 250 °C. ^1^H NMR (400 MHz, DMSO-*d*_6_) δ 7.33 (d, *J* = 8.6 Hz, 2H, Ar), 6.91 (d, *J* = 8.6 Hz, 2H, Ar), 6.02 (brs, 2H, -NH_2_), 5.92 (brs, 2H, -NH_2_), 5.12 (s, 2H, -CH_2_-), 5.07 (s, 1H, H5), 3.74 (s, 3H, -OCH_3_) ppm. ^13^C NMR (125 MHz, DMSO-*d*_6_) δ 169.54 (C_1′_), 165.05 (C_2_), 164.53 (C_4_), 159.38 (C_4′_), 130.66 (C_1′_), 129.37 (C_2′,6′_), 113.89 (C_3′,5′_), 81.01 (C_5_), 69.57 (-CH_2_-), 55.35 (C_4′_) ppm. Elemental analysis calcd (%) for C_12_H_14_N_4_O_2_: C, 58.53; H, 5.73; N, 22.75; found C, 58.59; H, 5.70; N, 22.75.

#### 3.2.7. Synthesis of Minoxidil Derivatives **55**–**58**

General procedure:

To a solution of the corresponding 6-substituted 2,4-diaminopyrimidine intermediate (1.16 mmol, 1 equiv.) in MeOH (3 mL), a solution of mCPBA (2 equiv.) in MeOH (5 mL) is added dropwise over a time period of 30 min at 0 °C. The reaction mixture is stirred at 0 °C for an additional 4–16 h. Then, aq. NaOH 4 N is added until a basic pH is reached. The organic solvent is evaporated under vacuum and the formed white solid precipitate is filtered under vacuum and washed with ice-cooled water (1 mL). The aqueous filtrate is extracted with EtOAc (3 × 50 mL). The combined organic layers are washed with brine (3 × 50 mL), dried over anh. Na_2_SO_4_, filtered, and concentrated. The resulting crude oil is purified by flash column chromatography (EtOAc/MeOH 0 to 30%), to afford an oil which is treated with Et_2_O under ice to afford the product as a white solid.

The compound 2,6-Diamino-4-(3-methoxyphenoxy)-1,6-dihydropyrimidine 1-oxide (**55**) was synthesized from the compound 51 (1 equiv.) according to the general procedure with a reaction time of 16 h. White solid (217.6 mg, 41%). R*_f_* = 0.27 (3:1 AcOEt: MeOH), m.p. > 250 °C (dec.: 200 °C). ^1^H NMR (500 MHz, DMSO) δ 7.31 (t, *J* = 8.1 Hz, 2H, OH, Ph), 6.79 (ddd, *J* = 8.4, 2.5, 0.9 Hz, 1H, Ph), 6.73–6.67 (m, 2H, Ph), 5.47 (s, 1H, CHC=NH), 3.75 (s, 3H, OCH_3_). ^13^C NMR (126 MHz, DMSO) δ 160.46 (C_4_), 159.35 (C_6_), 154.52 (C_1′_), 154.21 (C_2_), 152.67 (C_5′_), 113.22 (C_6′_), 110.71 (C_4′_), 107.10 (C_2′_), 77.49 (C_5_), 55.38 (OCH_3_). Elemental analysis calcd (%) for C_11_H_12_N_4_O_3_: C, 53.22; H, 4.87; N, 22.57. Found: C, 53.26; H, 4.90; N, 22.60.

The compound 2,6-Diamino-4-(2′,4′,5′-trichloro)-1,6-dihydropyrimidine 1-oxide (**56**) was synthesized from the compound 52 (1 equiv.) according to the general procedure with a reaction time of 16 h. White solid (120.6 mg, 41%). R*_f_* = 0.25 (3:1 AcOEt: MeOH), m.p. 135 °C. ^1^H NMR (400 MHz, DMSO) δ 8.02 (s, 1H, Ph), 7.79 (s, 1H, Ph), 7.46–7.30 (broad m, 3H, OH, NH_2_/NH), 5.65 (s, 1H, CHC=NH). ^13^C NMR (101 MHz, DMSO) δ 157.87 (C_4_), 154.35 (C_6_), 152.45 (C_2_), 150.00 (C_1′_), 148.32 (C_3′_, C_5′_), 131.11 (C_4′_), 130.53 (C_2′_), 125.81 (C_6′_), 77.11 (C_5_). Elemental analysis calcd (%) for C_10_H_7_Cl_3_N_4_O_2_: C, 37.35; H, 2.19; N, 17.42. Found: C, 37.39; H, 2.23; N, 17.46.

The compound 2,6-Diamino-4-(2′,4′-dichloro)-1,6-dihydropyrimidine 1-oxide (**57**) was synthesized from the compound **53** (1 equiv.) according to the general procedure with a reaction time of 16 h. White solid (169.5 mg, 45%). R*_f_* = 0.28 (3:1 AcOEt: MeOH), m.p. 160 °C. ^1^H NMR (400 MHz, DMSO) δ 7.82–7.83 (broad m, 1H, OH), 7.76 (d, *J* = 2.5 Hz, 1H, Ph), 7.48 (dd, *J* = 8.7, 2.5 Hz, 1H, Ph), 7.36 (d, *J* = 8.7 Hz, 1H, Ph), 5.59 (s, 1H, CHC=NH). ^13^C NMR (101 MHz, DMSO) δ 158.28 (C_4_), 154.31 (C_6_), 152.48 (C_2_), 147.96 (C_1′_), 130.00 (C_3′_), 129.80 (C_4′_), 128.60 (C_5′_), 127.38 (C_2′_), 125.55 (C_6′_), 76.89 (C_5_). HRMS/ESI+ (*m*/*z*): Calcd for C_10_H_9_ Cl_2_N_4_O_2_: 287,0103; Found: 287.0098. Elemental analysis calcd (%) for C_10_H_8_Cl_2_N_4_O_2_: C, 41.84; H, 2.81; N, 19.52. Found: C, 41.88; H, 2.85; N, 19.56.

The compound 2,6-Diamino-4-((4-methoxybenzyl)oxy)pyrimidine 1-oxide (**58**) was synthesized from the compound **54** (300.0 mg, 1.22 mmol) according to the general procedure with a reaction time of 4 h. White solid (17.8 mg, 6%). R*_f_* = 0.25 (EtOAc/MeOH 3:1), m.p. > 250 °C. ^1^H NMR (600 MHz, DMSO-*d*_6_) δ 7.34 (d, *J* = 8.6 Hz, 2H, Ar), 7.18 (br, s, *J* = 38.1 Hz, 4H), 6.92 (d, *J* = 8.7 Hz, 2H, Ar), 5.45 (s, 1H, H_3_), 5.11 (s, 2H, -CH_2_-), 3.75 (s, 3H, -OCH_3_) ppm. ^13^C NMR (101 MHz, DMSO-*d*_6_) δ 158.34 (C_2_), 155.36 (C_6_), 140.76 (C_4′_), 136.94 (C_4_), 129.37 (C_1′_), 127.32 (C_3′,5′_), 126.25 (C_2′,6′_), 97.88 (-CH_2_) 79.67 (-OCH_3_-), 67.18 (C_3_) ppm. Elemental analysis calcd (%) for C_12_H_14_N_4_O_3_: C, 54.96; H, 5.38; N, 21.36; found: C, 54.99; H, 5.42; N, 21.39.

#### 3.2.8. Synthesis of 5-Acetyl Barbituric Acid

Barbituric acid (1.0 g, 7.81 mmol) was suspended in acetic anhydride (23.4 mL) and 7 drops of conc. H_2_SO_4_ were added. After 10 min, the barbituric acid was completely dissolved, giving a yellow-brown solution. The reaction mixture was stirred at 110 °C for 1.5 h. Thereafter, the mixture was concentrated to half its volume and cooled down to 0 °C. The formed precipitate was filtered off and washed with hot water and acetone. Beige crystalline solid (1.15 g, 89%). ^1^H NMR (400 MHz, DMSO) δ 11.77 (s, 1H), 11.05 (s, 1H), 2.58 (s, 3H) ppm [49].

#### 3.2.9. Synthesis of Barbituric Acid Analogs **59**–**60**

The compound 5-Acetyl-barbituric acid (1 equiv.) is suspended in abs. EtOH (5 mL) at ~90 °C and molecular sieves and the appropriate hydroxylamine (1.1 equiv.) are added. The mixture is stirred for 3–4 days at 70 °C under an argon atmosphere. After 4 days, the reaction mixture color remains unchanged, and the reagents do not seem to dissolve. Moreover, TLC does not provide a clear image of whether the reaction has progressed. The suspended solid is slowly filtered under vacuum and washed with Et_2_O and EtOH. The molecular sieves are removed, to afford the desired product as a beige solid.

The compound Methyl 4′-((((1-(2,4,6-Trioxohexahydropyrimidin-5-yl)ethylidene)amino)oxy)methyl)benzoate (**59**) was synthesized from the compound 27 (1.1 equiv.) according to the general procedure. Beige-brown solid (195 mg, 94.8%). R*_f_* = 0.20 (AcOEt), m.p. > 250 °C. ^1^H NMR (500 MHz, DMSO) δ 10.47 (s, 1H, -NH), 10.21 (s, 1H, -NH), 8.92 (d, *J* = 8.2 Hz, 1H, H_5_), 7.95–7.88 (m, 1H, Ar), 7.86 (d, *J* = 8.2 Hz, 1H, Ar), 7.62 (d, *J* = 8.1 Hz, 1H, Ar), 7.54–7.50 (m, 1H, Ar), 5.01 (s, *J* = 40.5 Hz, 2H, OCH_2_), 3.84 (s, 3H, OCH_3_), 1.88 (s, *J* = 13.5 Hz, 3H, CH_3_). ^13^C NMR (75 MHz, DMSO) δ 170.7 (C_4_, C_6_), 165.9 (C-O), 164.6 (C_7_), 150.4 (C_2_), 141.6 (C_1′_), 130.1 (C_3′_, C_5′_), 129.3 (C_2′_, C_6′_), 129.0 (C_4′_), 76.9 (OCH_2_), 51.5 (C_5_), 14.4 (CH_3_). Elemental analysis calcd (%) for C_15_H_15_N_3_O_6_: C, 54.05; H, 4.54; N, 12.61. Found: C, 54.07; H, 4.56; N, 12.63.

The compound Methyl 2′-chloro-4′-((((1-(2,4,6-trioxohexahydropyrimidin-5-yl)ethylidene)amino)oxy)methyl)benzoate (**60**) was synthesized from the compound **23** (1.1 equiv.) according to the general procedure. Beige-brown solid (195 mg, 85.3%). R*_f_* = 0.20 (AcOEt), m.p. > 250 °C. ^1^H NMR (500 MHz, DMSO) δ 10.47 (s, 1H, -NH), 10.21 (s, 1H, -NH), 8.92 (d, *J* = 8.2 Hz, 1H, H_5_), 7.95–7.88 (m, 1H, Ar), 7.86 (d, *J* = 8.2 Hz, 1H, Ar), 7.62 (d, *J* = 8.1 Hz, 1H, Ar), 5.01 (s, *J* = 40.5 Hz, 2H, OCH_2_), 3.84 (s, 3H, OCH_3_), 1.88 (s, *J* = 13.5 Hz, 3H, CH_3_). ^13^C NMR (75 MHz, DMSO) δ 170.7 (C_4_, C_6_), 165.9 (C-O), 164.6 (C_7_), 150.4 (C_2_), 142.5 (C_1′_), 133.4 (C_4′_), 132.3 (C_2′_), 130.1 (C_3′_), 128.4 (C_6′_), 128.2 (C_5′_), 76.9 (OCH_2_), 51.5 (C_5_), 14.4 (CH_3_). Elemental analysis calcd (%) for C_15_H_14_ClN_3_O_6_: C, 48.99; H, 3.84; N, 11.43. Found: C, 49.00; H, 3.86; N, 11.45.

### 3.3. Cells and Cell Culture

HepDES19 cells were incubated on collagen-coated plates at 37 °C with 5% CO_2_ and saturating humidity in Dulbecco’s modified Eagle’s medium (DMEM/F12) (Cytiva Life Sciences, Marlborough, MA, USA) supplemented with 10% fetal bovine serum (FBS), penicillin (100 IU/mL), and streptomycin (100 µg/mL). HepDES19 cells are HepG2 cells that carry a stably transfected HBV genotype D genome under the control of a tetracycline repressible promoter [50]. Cells were maintained in the presence of 1 µg/mL tetracycline to repress the expression of the stably integrated HBV genome, and HBV replication was induced by the withdrawal of tetracycline from the culture medium.

### 3.4. qPCR HBV Replication Inhibition Assay

HBV replication inhibition was measured in HepDES19 cells induced to replicate HBV by the removal of tetracycline using a strand-preferential quantitative PCR assay as previously described [29,51]. Cells were seeded at 4 × 10^4^ cells/well in 96-well plates for 48 h. Serially diluted compound was then added to cells at a final concentration of 1% DMSO for 72 h, after which the cells were lysed and qPCR was performed as described in Li et al. [51]. EC_50_ values were calculated from the (+) DNA data with GraphPad Prism using the four-parameter log (inhibitor) versus response algorithm with the bottom value set to zero. Three or more replicate assays were performed on different days. 

### 3.5. MTS Cytotoxicity Assay

Cell viability was assessed using the CellTiter 96^TM^ Aqueous Non-Radioactive Cell Proliferation assay (MTS) (Promega, Madison, WI, USA) as described previously [29]. HepDES19 cells were seeded at 1 × 10^4^ cells per well in 96-well, and compound was added after 48 h. Cells incubated with serially diluted compound (1% DMSO) for 72 h. Bac absorbance values were subtracted, and data were converted to percent cell viability. The cytotoxic concentration 50% (CC_50_) values were calculated with GraphPad Prism by using the four-parameter variable response log (inhibitor) versus response algorithm with the bottom value set to zero. Three or more replicate assays were performed on different days.

### 3.6. Solubility Limit Assay

Compound solubility limits were tested in DMEM-F12 without phenol red (Gibco, Grand Island, NY, USA) supplemented with 10% FBS (pH 7.2–7.4) to mimic cell culture experiments, as we have performed previously [29]. Compounds were serially diluted in buffer at pH 7.4 in 384-well, optically clear plates (upper limit 200 µM, DMSO 1%) and read on a plate reader at 620 nm. Compound concentration (µM) was plotted against optical density (OD) and a solubility limit was determined by identifying an inflection point where there was a significant increase in optical density due to increasing turbidity; the concentration of the compound at the inflection point was defined as the solubility limit. Two or more replicate assays were performed on different days for each compound.

### 3.7. Parallel Artificial Membrane Permeability Assay

Apparent passive permeability (P_app_) was assessed at pH 7.4 using a 96-well donor/acceptor cassette (Sigma Aldrich, Burlington, MA, USA), which mimics the apical and basolateral sides of the small intestine epithelium, as we have described previously [30]. Briefly, an artificial membrane composed of 1% *w/v* lethicin/dodecane was added to the poly (vinylidene fluoride) (PVDF) membrane filter. Once dry, compounds (200 μM) were diluted in buffer at pH 7.4 and added to the donor side of the membrane, and the same pH buffer was added to the acceptor side. The cassette was assembled and incubated at room temperature with shaking at 250 rpm for 2 h, after which 100 μL of the acceptor well was retrieved and the experimental compound absorbance was read on a plate reader between 200 and 600 nm. Compound P_app_s (cm/s) values were determined by normalizing to the compound absorbance at equilibrium, incubation time, and membrane porosity Two or more replicate assays were conducted on different days.

### 3.8. Molecular Modeling

The full-length HBV P model used in docking studies was generated through Alphafold2 and then prepared by placing Mg^+2^ ions into the active site of the RNase H domain by superposition of the DEDD active site motif of RNase H onto the cocrystal structure of HIV RNase H (PDB: 3K2P) [18]. A PDB file for the HBV model used for docking can be found in [18]. The Ligprep and Protein Preparation Wizard programs in the Schrödinger suite (Schrödinger LLC, New York, NY, USA) were used to prepare ligands and the protein, respectively, as mentioned in Giannakopoulou et al. [30]. Thirty-six conformers of each ligand were generated by the Ligprep routine and used to seed the docking algorithm. The protonation states of ligands and the protein structures were given at pH 7.5 ± 2 and were energy minimized with an OPSL4 force field. The induced fit docking (IFD) program of the Schrödinger suite (Schrödinger LLC) that allows both the ligand and protein to change shape to minimize the energy during binding was used to analyze the binding poses of HPDs, as described previously [30]. A receptor grid of 10 Å was used to dock the compounds into the active site of the HBV RH domain. Refinement of the protein–ligand complex was performed using a van der Waals radius scaling of 0.5. Residues were refined within 5 Å of the ligand poses of the top 20 structures after initial docking. Redocking was performed with the top structures within a 30 kcal/mol energy window using Glide XP precision (Schrödinger LLC, New York, NY, USA, Release 2023-3).

## 4. Conclusions

This work represents part of our ongoing project of creating safe and effective HBV RNase H inhibitors. In total, 18 new HPD oximes plus 4 structurally related minoxidil and 2 barbituric acid analogs were designed, synthesized, and evaluated for their ability to inhibit HBV replication. All of the HPD oximes were active against HBV while exhibiting minimal cytotoxicity. Additionally, they had high rates of apparent passive permeability and had high solubility limits at pH 7.4. The oxime group appears to be the better choice for linking the main HPD ring to the side aromatic segment because the HPD oximes have more promising profiles than corresponding HPD imines in both in vitro and in silico studies. The benzyl moiety with small lipophilic substituents (especially in the 4′-position) appears to be the best of the tested side aromatic components, whereas adding extra aromatic rings or altering the linker group does not seem to improve the compound’s performance. The minoxidil and barbituric acid analogs, while promising in computational studies, did not exhibit any antiviral activity. The results of this study provide deeper insights into the SARs of the HPD compound class and reinforce HBV RNase H as a promising drug target for combating HBV. These findings will also guide the further optimization of the HPD scaffold in our ongoing efforts to develop more effective and selective HBV RNase H inhibitors. Future research will aim to further optimize the side aromatic moiety of HPDs and explore the incorporation of alternative pharmacophore rings with metal-binding groups capable of chelating Mg^2+^ ions in the enzyme’s RNase H active site.

## Figures and Tables

**Table 1:** Compound structures, physical properties, efficacy, and cytotoxicity.

a/a	Structure	logP ^1^	logD ^2^	tPSA ^3^	Fsp3 ^4^	Docking Score ^5^	EC_50_ ^6^	CC_50_ ^6^	SI ^7^
**33**	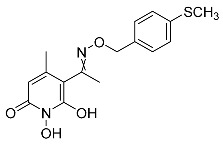	1.7	1	84	0.25	−9.4	1.4 ± 0.7	100 ± 0	71.6
**34**	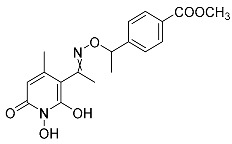	1.8	1.5	110.4	0.28	−9.6	6.3 ± 5.5	96.0 ± 5.7	15.3
**35**	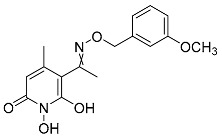	1.1	0.4	93.3	0.25	−10.03	1.9 ± 1.2	90.0 ± 14.1	47.0
**36**	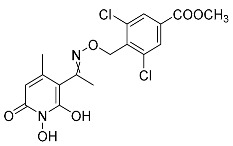	2.2	1.6	110.4	0.24	−10.5	3.1 ± 0.4	100.0 ± 0	32.4
**37**	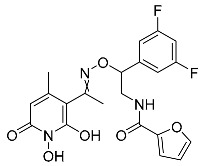	1.9	1.2	126.3	0.19	−10.0	4.7 ± 3.0	100 ± 0	21.5
**38**	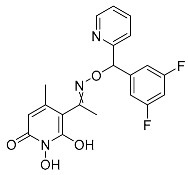	1.5	1.4	96.9	0.15	−10.1	2.3 ± 0.2	47.1 ± 21.7	20.5
**39**	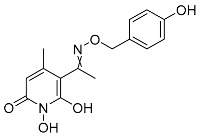	0.5	−0.1	104.3	0.2	−11.1	5.2 ± 0.9	91.1 ± 14.3	17.7
**40**	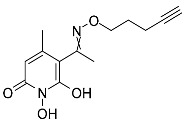	0.1	−0.6	84	0.38	−9.4	2.5 ± 0.6	53.6 ± 31.4	21.6
**41**	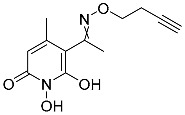	−0.4	−0.9	84	0.33	−9.5	1.2 ± 0.2	57.5 ± 32.4	47.7
**42**	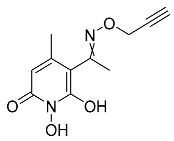	−0.4	−0.8	84	0.27	−8.9	5.4 ± 2.2	81.7 ± 18.6	15.1
**43**	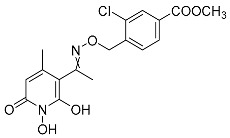	2.1	1.1	110.4	0.24	−10.6	5.5 ± 2.0	83.8 ± 11.8	15.1
**44**	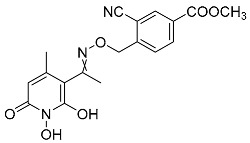	1.2	0.3	134.1	0.22	−10.3	7.7 ± 1.4	91.9 ± 11.2	11.9
**45**	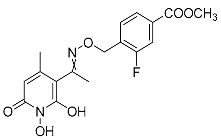	1.5	0.6	110.4	0.24	−10.8	3.2 ± 0.4	90.2 ± 13.8	28.4
**46**	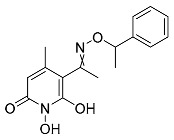	1.6	1.4	84	0.25	−9.8	3.1 ± 0.5	100.0 ± 0	32.7
**47**	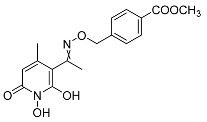	1.2	0.4	110.4	0.24	−10.9	1.1 ± 0.4	100.0 ± 0	87.9
**48**	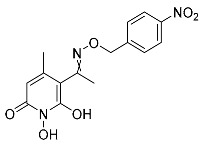	1.1	0.5	127.2	0.2	−9.7	1.1 ± 0.2	99.6 ± 0.6	91.7
**49**	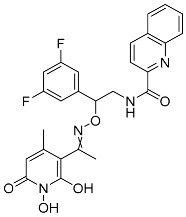	3.4	2.5	126	0.15	−9.7	3.5 ± 0.8	99.9 ± 0.2	28.9
**50**	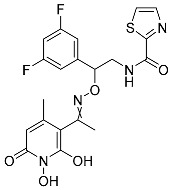	2.7	1.1	126	0.2	−9.5	4.0 ± 1.5	100.0 ± 0	25.1
**55**	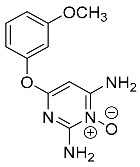	0.2	0.2	106.4	0.09	−8.8	-	-	-
**56**	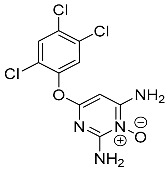	1.9	1.9	94.2	0	−8.2	-	-	-
**57**	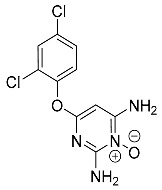	1.2	1.2	97.2	0	−8.18	-	-	-
**58**	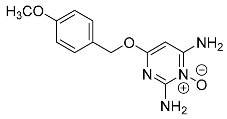	0.2	0.2	106.4	0.17	−8.17	-	-	-
**59**	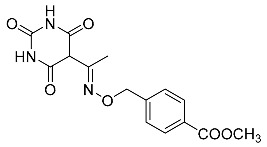	1.2	0.9	123.2	0.27	−5.7	-	-	-
**60**	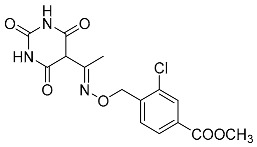	2.1	1.6	123.2	0.27	−5.5	-	-	-

^1^ LogP (<5) is the log of the partition coefficient of a solute between octanol and water. Predicted with the FAF4 online server [34]. ^2^ LogD is the log of the partition coefficient of a solute between 1-octanol and water at pH 7.4, or physiological pH. ^3^ tPSA (<140 Å^2^) is the topological polar surface area (Å^2^). ^4^ Fsp^3^, the number of sp^3^ hybridized carbons/total carbon count. ^5^ Induced fit docking score to the HBV RNase H active site; kCal/Mol. ^6^ pH 7.4; values in µM. ^7^ CC_50_/EC_50_.

**Table 2:** Compound solubility limits and apparent passive permeability at pH 7.4.

	Solubility Limit ^1^	P_app_ ^2^	
Compound No.	-	cm/s	Interpretation
**33**	200	4.35 × 10^−6^	H
**34**	50	2.50 × 10^−6^	H
**35**	117	6.19 × 10^−6^	H
**36**	163	4.35 × 10^−6^	H
**37**	175	4.96 × 10^−6^	H
**38**	167	2.61 × 10^−6^	H
**39**	200	5.21 × 10^−6^	H
**40**	200	3.36 × 10^−6^	H
**41**	200	2.24 × 10^−6^	H
**42**	167	3.74 × 10^−6^	H
**43**	200	2.21 × 10^−6^	H
**44**	200	5.42 × 10^−6^	H
**45**	200	3.54 × 10^−6^	H
**46**	200	3.19 × 10^−6^	H
**47**	200	5.52 × 10^−6^	H
**48**	200	5.38 × 10^−6^	H
**49**	200	6.71 × 10^−6^	H
**50**	200	4.67 × 10^−6^	H
**55**	200	6.17 × 10^−6^	H
**56**	167	3.85 × 10^−6^	H
**57**	100	2.99 × 10^−6^	H
**58**	200	2.56 × 10^−6^	H
**59**	INS	INS	INS
**60**	INS	INS	INS

^1^ Values are in µM. ^2^ H: High rate of apparent passive permeability (>1 × 10^−6^ cm/s); L: Low rate of apparent passive permeability (<1 × 10^−6^ cm/s). INS: Compound was insoluble in 100% DMSO.

## Data Availability

Data are contained within the article and Supplementary Materials.

## Supplementary Materials

The following supporting information can be downloaded at: https://www.mdpi.com/article/10.3390/molecules29122942/s1, Table S1: Selection of calculated druglike properties for the tested compounds.
