# Supplementary material for: N-Hydroxypiridinedione: A Privileged Heterocycle for Targeting the HBV RNase H
Source: Molecules. 2024 Jun 20;29(12):2942. doi: 10.3390/molecules29122942 (PMC11206691; doi:10.3390/molecules29122942)
Supplement: Supplementary file 1 [file molecules-29-02942-s001.zip › molecules-3057531-supplementary.pdf]

Supporting Information

N-hydroxypiridinedione: a privileged heterocycle for targeting the HBV RNase H

Dimitrios Moianos [1], Maria Makri [1], Georgia-Myrto Prifti [1], Aristeidis Chiotellis [2], Alexandros Pappas [2], Molly E. Woodson [3], Razia Tajwar [3], John E. Tavis [3] and Grigoris Zoidis [1,*]

[1] Department of Pharmacy, Division of Pharmaceutical Chemistry, School of Health Sciences, National and Kapodistrian University of Athens, Panepistimiopolis
Zografou, 15771 Athens, Greece; moianosjim@gmail.com (D.M.); mariamakri1@hotmail.com (M.M.); myrtoprifti@gmail.com (G.-M.P.)
[2] Institute of Nuclear & Radiological Sciences & Technology, Energy & Safety, National Center for Scientific Research "Demokritos", 15310 Athens, Greece; achiotel@rrp.demokritos.gr (A.C.); alpappas@pharm.uoa.gr (A.P.)
[3] Molecular Microbiology and Immunology, Saint Louis University School of Medicine, Saint Louis, MO 63104, United States; molly.woodson@slu.edu (M.W.); razia.tajwar@health.slu.edu (R.T.); john.tavis@health.slu.edu (J.E.T.)
* Correspondence: Prof. Grigoris Zoidis (zoidis@pharm.uoa.gr), Tel.: +302107274809

Table S1: Selection of calculated Drug-like properties for the tested compounds.

Cmpd	dipole	SASA	FOSA	FISA	PISA	volume	QPpolrz	QPlogPo/w	QPlogS	CIQPlogS	QPPCaco	QPlogBB	QPPMDCK	QPlogKp	% Human Oral Absorption	PSA	Rule of 5	Rule of 3
**33**	6.385	588.140	242.458	157.293	148.508	1043.165	31.875	1.924	-3.335	-3.817	319.367	-1.287	238.240	-3.127	83.032	93.417	0	0
**34**	7.939	645.944	297.021	210.145	138.779	1146.719	35.926	1.415	-3.640	-3.762	100.716	-2.001	41.383	-4.136	71.080	128.602	0	0
**35**	4.760	610.001	263.435	157.592	188.973	1043.759	32.285	1.697	-3.438	-3.426	317.290	-1.497	143.049	-2.990	81.651	102.835	0	0
**36**	6.346	689.436	251.483	212.887	115.831	1203.756	37.990	2.103	-4.817	-4.814	94.862	-1.903	153.852	-4.267	74.646	131.006	0	0
**37**	7.446	675.352	149.771	190.581	251.957	1256.625	40.121	2.191	-3.899	-5.323	154.392	-1.663	187.178	-3.184	78.949	135.223	0	1
**38**	6.990	672.266	136.069	146.289	298.252	1205.790	39.813	3.138	-4.775	-5.282	406.111	-1.162	593.517	-2.397	92.010	102.974	0	1
**39**	5.837	584.100	170.480	212.345	201.275	990.500	30.271	0.904	-3.041	-3.157	95.991	-2.010	39.288	-3.956	67.718	117.187	0	0
**40**	4.981	564.088	301.518	159.659	102.911	931.205	26.317	1.134	-2.758	-2.342	303.284	-1.571	136.236	-3.235	78.004	94.753	0	0
**41**	5.467	521.275	260.641	149.238	111.396	857.500	24.090	0.805	-2.268	-2.101	380.778	-1.338	174.223	-3.109	77.850	88.397	0	0
**42**	5.075	493.118	221.617	152.803	118.698	798.221	22.430	0.456	-2.017	-1.867	352.267	-1.289	160.167	-3.245	75.197	88.765	0	0
**43**	4.411	669.913	258.422	215.689	149.730	1160.842	36.596	1.626	-4.225	-4.150	89.232	-2.067	64.919	-4.199	71.377	130.708	0	0
**44**	4.863	640.555	240.526	268.208	131.821	1139.707	34.937	0.383	-3.937	-4.414	28.345	-2.614	10.512	-5.134	55.182	155.160	0	0
**45**	4.863	671.405	265.289	215.827	152.625	1147.220	36.078	1.506	-4.213	-3.837	88.964	-2.135	58.197	-4.191	70.649	131.024	0	0
**46**	8.419	585.176	213.017	146.944	225.216	1013.755	32.073	1.935	-3.418	-3.392	400.341	-1.267	183.918	-2.762	84.855	92.125	0	0
**47**	7.284	665.041	269.470	215.719	179.852	1132.773	35.760	1.308	-3.946	-3.499	89.173	-2.222	36.281	-4.094	69.512	130.927	0	0
**48**	2.018	611.826	171.071	255.194	185.561	1041.319	32.155	0.903	-3.395	-3.617	37.662	-2.499	14.291	-4.801	60.436	139.549	0	0
**49**	7.833	774.792	143.008	204.777	342.282	1449.558	48.705	3.126	-5.315	-6.617	113.240	-1.967	136.761	-3.128	69.051	136.966	1	1
**50**	8.221	701.719	123.168	208.465	239.199	1289.342	41.309	2.029	-4.235	-5.279	104.479	-1.803	224.411	-3.559	74.962	140.740	0	1
**55**	1.608	453.040	90.924	170.277	191.839	763.187	23.010	0.883	-2.032	-3.109	240.528	-1.166	106.039	-3.502	74.735	101.413	0	0
**56**	1.461	482.890	0.000	167.275	129.430	809.987	24.929	2.023	-3.538	-4.707	256.822	-0.693	1191.667	-3.762	81.916	91.961	0	0
**57**	3.245	462.537	0.000	167.198	167.466	769.717	23.681	1.611	-2.962	-4.040	257.255	-0.809	572.147	-3.627	79.518	91.937	0	0
**58**	3.074	512.998	125.994	168.739	218.265	845.161	25.901	1.519	-2.853	-3.500	248.740	-1.382	109.957	-3.284	78.721	99.534	0	0
**59**	5.405	572.537	183.052	251.998	137.487	991.253	30.976	0.673	-3.156	-3.688	40.384	-2.181	15.411	-5.103	59.634	166.113	0	0
**60**	3.950	583.766	175.262	250.369	114.217	1021.252	31.955	1.004	-3.524	-4.345	41.846	-2.055	27.868	-5.155	61.849	166.226	0	0

The above table is a selection of properties modelled with QikProp – Schrödinger. A brief description of all QikProp calculated properties and descriptors can be found below.

Properties/descriptors calculated by QikProp

dipole Computed dipole moment of the molecule (D)
SASA Total solvent accessible surface area (SASA) in square angstroms using a probe with a 1.4 Å radius
FOSA Hydrophobic component of the SASA (saturated carbon and attached hydrogen)
FISA Hydrophilic component of the SASA (SASA on N, O, and H on heteroatoms)
PISA π (carbon and attached hydrogen) component of the SASA
WPSA Weakly polar component of the SASA (halogens, P, and S)
volume Total solvent-accessible volume in cubic angstroms using a probe with a 1.4 Å radius
QPpolrz Predicted polarizability in cubic angstroms
QPlogPo/w Predicted octanol/water partition coefficient
QPlogS Predicted aqueous solubility
CIQPlogS Conformation-independent predicted aqueous solubility
QPPCaco Predicted apparent Caco-2 cell permeability in nm/sec
QPlogBB Predicted brain/blood partition coefficient
QPPMDCK Predicted apparent Madin-Darby canine kidney cell permeability in nm/sec
QPlogKp Predicted skin permeability
PercentHumanOralAbsorption Predicted human oral absorption on 0 to 100% scale
PSA Van der Waals surface area of polar nitrogen and oxygen atoms
Rule Of Five Number of violations of Lipinski's rule of five
Rule Of Three Number of violations of Jorgensen's rule of three

Materials & Methods:
The 3D structures were generated in Maestro from the respective SMILES strings, and chiral centers were set accordingly where necessary. The structures were minimized in neutral state using Macromodel with the settings previously described. The minimized structures were used as input in QikProp under default settings.

Schrödinger Release 2024-1: QikProp v7.8, Schrödinger, LLC, New York, NY, 2024.
